# Interfacial and Crystalline *Gauche* OCH_2_–CH_2_O Layers in Biodegradable and Recyclable Polyethylene-like Polyesters Detected by Nuclear Magnetic Resonance

**DOI:** 10.1021/jacs.5c10528

**Published:** 2025-12-02

**Authors:** Zhenhuan Sun, Taylor Frederick Nelson, Stefan Mecking, Klaus Schmidt-Rohr

**Affiliations:** † Department of Chemistry, 8244 Brandeis University, Waltham, Massachusetts 02453, United States; ‡ Department of Chemistry, 26567 University of Konstanz, 78457 Konstanz, Germany

## Abstract

Polyethylene-like aliphatic polyesters are promising biodegradable polymers; however, their conformational and supramolecular structures are not well understood. Here, we used solid-state nuclear magnetic resonance (NMR) to investigate three synthetically accessible polyesters made from doubly ^13^C-labeled ethylene diol units and unlabeled dicarboxylic acids of 12-, 18-, and 48-carbon length (PE-2,12 to PE-2,48). Signals of abundant *gauche* OCH_2_–CH_2_O conformers observed in all samples are spectrally resolved from the sharp peak of crystalline *anti* OCH_2_-CH_2_O segments in PE-2,12 and PE-2,18. Layers of disordered and immobilized *gauche* OCH_2_–CH_2_O units at the crystal–amorphous interfaces are present in all samples. PE-2,12 and PE-2,18 additionally contain mobile amorphous and crystalline *gauche* OCH_2_-CH_2_O units. The unexpected crystalline *gauche* conformation deduced from the chemical shift and slow ^13^C spin–lattice relaxation was proved by fast decay in centerband-only detection of exchange (CODEX) NMR. The location of these *gauche* OCH_2_ groups deep inside the crystallites was confirmed by ^1^H spin diffusion from the amorphous layers. Crystalline *gauche* moieties, observed in three different samples of PE-2,12, account for about 1/3 of its crystalline OCH_2_ groups. Based on quantitative NMR and spin diffusion, specific models of the layered supramolecular structures were developed, with *gauche* OCH_2_ in interfacial layers at the crystal surfaces. While PE-2,18 and PE-2,12 contain two or three *anti* diol/diester layers within each crystallite, most OCH_2_ groups in PE-2,48 are immobilized at the interfaces, and mobile *gauche* or crystalline *anti* OCH_2_ units are insignificant. Thus, PE-2,48 contains all-polyethylene crystalline lamellae capped by diol/diester interfacial layers, indicating chemical control of the crystallite thickness.

## Graphical abstract

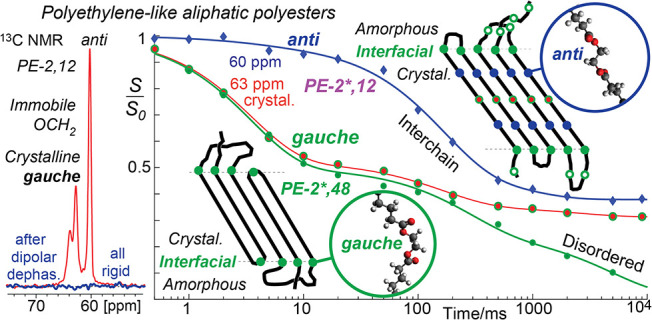

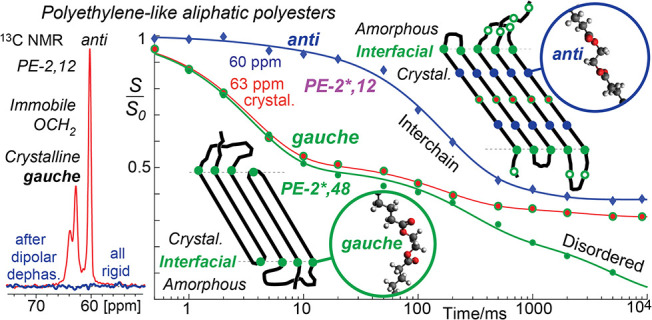

## Introduction

Plastics are essential components of all modern technologies, fulfilling a multitude of functions due to their low production costs and favorable properties as materials. As a downside, their production is based primarily on fossil fuel feedstocks,^1^ and their durability granted from hydrocarbon backbones translates to difficulties in efficient recycling,^2,3^ and renders them persistent when leaked from waste management streams into the environment.^4^ The large and growing scale of plastics production, in combination with their often short-lived applications,^5^ means that they are one of the major contributors to anthropogenic greenhouse gas emissions,^6^ and a major class of emerging environmental pollutants of concern.^7^ Therefore, sustainable polymers that can be derived from renewable feedstocks and enable circular end-of-life treatment options, while maintaining useful materials properties of traditional polymers, are of high and urgent interest.

A concept of such sustainable polymers as replacements for traditional ones has recently been showcased for the plastic of largest production volume, polyethylene (PE).^8,9^ Linear long-chain, aliphatic polyesters, see examples in Scheme 1, represent polyethylene chains via long stretches of repeating methylene units, while having a low concentration of in-chain ester groups. These functional groups act as breakpoints for chemical recycling under mild conditions and render the polymers amenable to biodegradation. At the same time, favorable PE-like mechanical properties and processability are maintained. This is due to the disperse inclusion of oxygen-containing functional groups into the polymer backbone, which does not compromise the polyethylene-like crystal structure, dominated by van der Waals interactions between hydrocarbon chains, as shown prominently via wide-angle X-ray scattering patterns matching that of high-density polyethylene.^8,10^ The ester moieties are located not only in the amorphous regions but can also be accommodated in the crystallites, as concluded from the polymers’ thermal properties, and studies of the morphologies via solid state nuclear magnetic resonance (NMR).^11^ For polyesters with isolated ester groups in the chains, generated from long-chain diols and long-chain dicarboxylates, monitoring of the crystallization by differential scanning calorimetry indicates formation of layered structures with multiple methylene run length (CH_2_)_*n*_ in a crystalline lamella, apparently even for a long run length of 48 carbon atoms.^12–14^

**Scheme 1:**
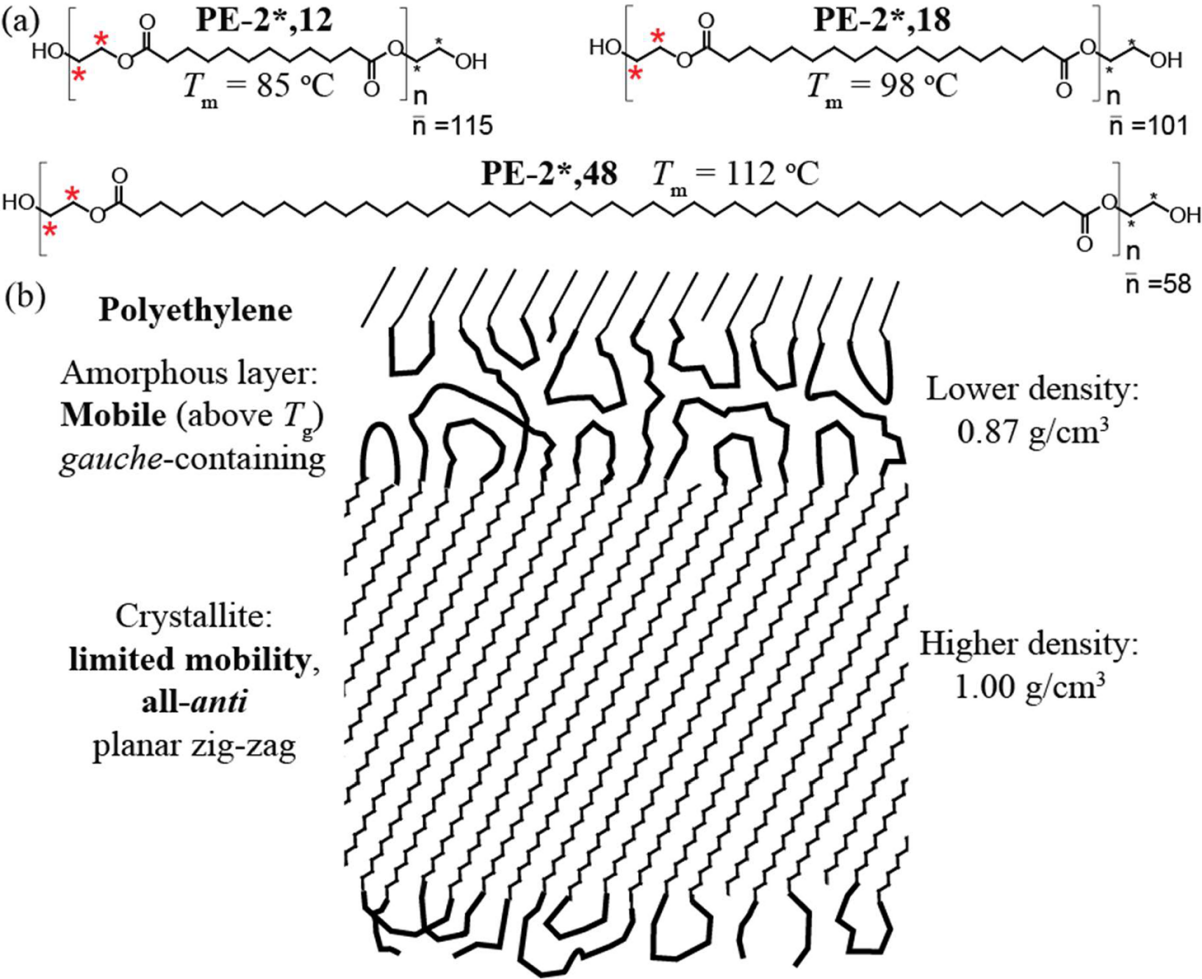
(a) Chemical Structures and Key Characteristics (*T*_m_ = Peak Melting Temperature; *n̅* = Average Degree of Polymerization) of the Degradable Polyethylene-like Polyesters Investigated in This Study^a^

a Asterisks mark ^13^C-labels. The chain-length dispersity indices D are between 2.5 and 3, and crystallinities range between 60% (PE-2*,12) and 76% (PE-2*,48; see Table S1). (b) Cartoon of the chemical structure of high-density polyethylene,^20,21^ with all-*anti* planar zigzag conformations in the wide lamellar crystallites (though realistically, the sequence of *anti* bonds is at least twice as long as shown) and large-amplitude mobility in the soft, less dense amorphous layers. Chain tilt and chains ending at the crystal surface avoid a density anomaly at the crystal–amorphous interface.^22^

Long-chain polyesters made from short-chain diols like ethylene glycol offer the benefit of commercial availability of their constituent monomers, while still retaining polyethylene-like properties.^9,15,16^ Short-chain diols are also available from renewable sources. Moreover, polyester-2,18, see Scheme 1a, has recently been found to be particularly amenable to biodegradation and thereby provides a perspective for nonpersistent polyethylene-like materials.^9,17^ The key to this material’s increased biodegradability can be hypothesized as the accessibility of the ester groups to microbial extracellular enzymes that catalyze depolymerization.^17^ What remains unclear is how the short-chain diol-derived adjacent double ester moieties adapt into a polyethylene-like solid and the structural consequences. Herein, we employ advanced solid state NMR methods to elucidate these fundamental issues by providing precise information on the conformation, dynamics, and morphological location of the diol units.

The ^13^C-labeled samples analyzed here were synthesized from commercially available ^13^C_2_-ethylene glycol and are clearly suitable for NMR studies of ester layering.^11^ In addition to the “canonical” polyester PE-2,18, we also characterize PE-2,12 and PE-2,48, see Scheme 1a, as materials with high and low ester concentration, respectively. OCH_2_ segments with ^13^C chemical shifts indicating *gauche* conformations are prominently observed, including a significant immobile fraction. This was unexpected because the chain conformation in the crystallites of PE is all-*anti*, see Scheme 1b, and X-ray diffraction shows similar chain packing in PE-2,N,^8,10^ while chains are highly dynamic in the amorphous layers.^22^ Nevertheless, such OCH_2_–CH_2_O *gauche* conformations are in line with the helical structure of crystalline poly­(ethylene oxide)^18^ and an NMR study^19^ of the ethylene glycol unit in amorphous poly­(ethylene terephthalate), PET, which showed a preference of OCH_2_–CH_2_O segments for *gauche* conformations, also known as the “anomeric effect”, when straight-chain-packing energetics are not dominant. To obtain selective spectra of different *gauche* (amorphous, interfacial, and crystalline) and *anti* components, systematic differences in ^13^C chemical shift, ^13^C spin–lattice relaxation, and ^13^C–^1^H dipolar couplings are utilized. Centerband-only detection of exchange (CODEX) ^13^C NMR is used to convincingly distinguish *anti* conformers, where both carbons have the same chemical shift, from immobile *gauche* conformers, where the instantaneous, anisotropic chemical shifts of the directly bonded ^13^C nuclei are distinctly different. Through ^1^H spin diffusion with ^13^C detection, the location of the *gauche* conformers in the layered supramolecular structure is established.

## Results

### Quantitative ^13^C NMR

Figure 1a compares the quantitative ^13^C NMR spectra of three polyesters containing ^13^C_2_-ethylene glycol (indicated by *) and nonlabeled dicarboxylates of C_12_, C_18_, or C_48_ length, i.e., PE-2*,12, PE-2*,18 and PE-2*,48, see Scheme 1a. Samples were prepared for ^13^C solid-state NMR analyses by melt-homogenizing in vacuo before cryo-milling to fine powders, unless otherwise indicated (see the SI for details). The spectra are dominated by O^13^CH_2_ signals between 60 and 68 ppm, enhanced ∼90-fold by the ^13^C-enrichment. Between 20 and 38 ppm, the signals of the carbons not bonded to oxygen, including PE-like (CH_2_)_*n*_ near 33 ppm, can be observed in natural abundance. The peaks of the ester carbons near 174 ppm are not shown here.

**Figure 1:**
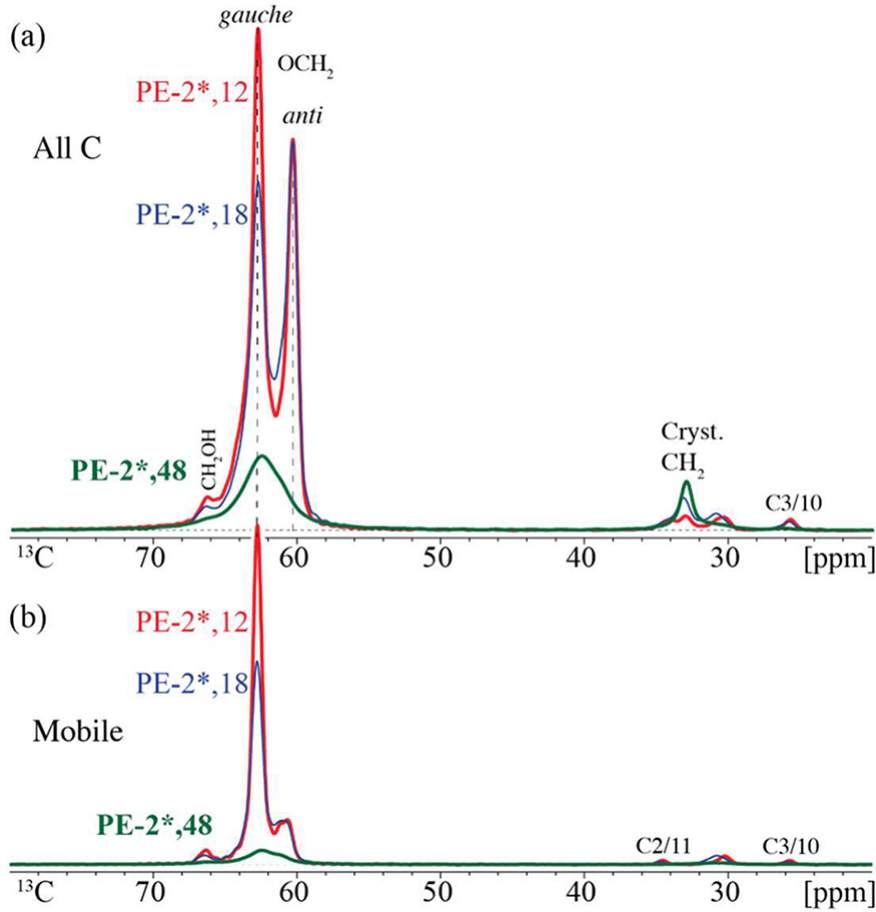
(a) Quantitative direct-polarization ^13^C NMR spectra of PE-2*,12, PE-2*,18, and PE-2*,48 scaled to similar area of the signals between 24 and 36 ppm. The total OCH_2_ signal of PE-2*,18 is expected to be 33% smaller, and that of PE-2*,48 four times smaller, than that of PE-2*,12. (b) Corresponding spectra of segments with fast large-amplitude mobility, obtained after recoupled dipolar dephasing with ^1^H decoupling switched off for 68 μs. C2/C11 and C3/10 refer to carbons of the diester segments.

The OCH_2_ spectra patterns of the three polyesters in Figure 1 are distinctly different. PE-2*,12 and PE-2*,18 both show two sharp peaks, but of significantly different relative intensities. Based on dipolar dephasing as a mobility filter passing only signals of CH_2_ groups with C–H dipolar couplings significantly averaged by large-amplitude motions, see Figure 1b, the left sharp peak at 62.5 ppm can be attributed to mobile segments in the amorphous layers. In the full spectra of Figure 1a, the small width of the peak of immobile segments near 60 ppm (fully dipolar dephasing in Figure 1b) indicates a uniform crystalline environment. The analogy with the all-*anti* conformation in the crystallites of PE suggests that the distinct conformation of these crystalline segments is *anti*. The two peak positions are in good agreement with the spectrum of the ethylene glycol segment in a classical polyester, polyethylene terephthalate, where the *gauche* and *anti* peaks are similarly observed near 63 and 60 ppm, respectively.^23^ The spectrum of PE-2*,48 shows predominantly a broad signal of *gauche* segments of limited mobility, as evidenced by their pronounced dipolar dephasing in Figure 1b. Due to their larger spacing by the relatively long dicarboxylate units, these O^13^CH_2_ units are more dilute and therefore have a lower signal compared to PE-2*,18. In all three samples, the relative intensity of the crystalline *anti* peak near 60 ppm is smaller than the crystallinity (i.e., the fraction of crystalline segments, 57–76%, see the SI with Table S1 and Figures S6–S8), quite dramatically so in PE-2*,12 and PE-2*,48. The reasons for this reduced *anti* signal, related to two different types of immobilized *gauche* segments, will be explained in the following.

### *T*_1C_ Relaxation of Immobile *Gauche* Components

Since the chain segments in PE crystallites are all-*anti* while they are highly mobile in the amorphous layers, see Scheme 1b, immobilized *gauche* segments were not anticipated when we started this study; exploring these novel components is a main focus of this work. We found, see the broad peaks near 62.5 ppm in Figure 2a,b, that such immobile *gauche* components can be selected by short CP and inverse dipolar dephasing (see Scheme S1c). Thus, Figure 2 shows series of ^13^C spectra of mostly immobile components, selected by short cross-polarization and inverse dipolar dephasing, in PE-2*,12 and PE-2*,18 with increasing *T*_1C_-filter time; the spectra after dipolar dephasing are included in Figure S1; corresponding less selective but more quantitative series of spectra after full direct polarization are shown in Figure S2. Notably, significant signals of immobile *gauche* components are detected near 62.5 ppm in Figure 2a,b and will be shown to arise significantly from segments at the crystal–amorphous interface. In PE-2*,18, more than half of these relax away within 1 s, leaving a relatively low, poorly resolved skewed background that nearly disappears within 5 s of relaxation; CODEX strongly indicates that the main component at ≤ 62.2 ppm is due to *anti* segments and therefore of little interest here.

**Figure 2:**
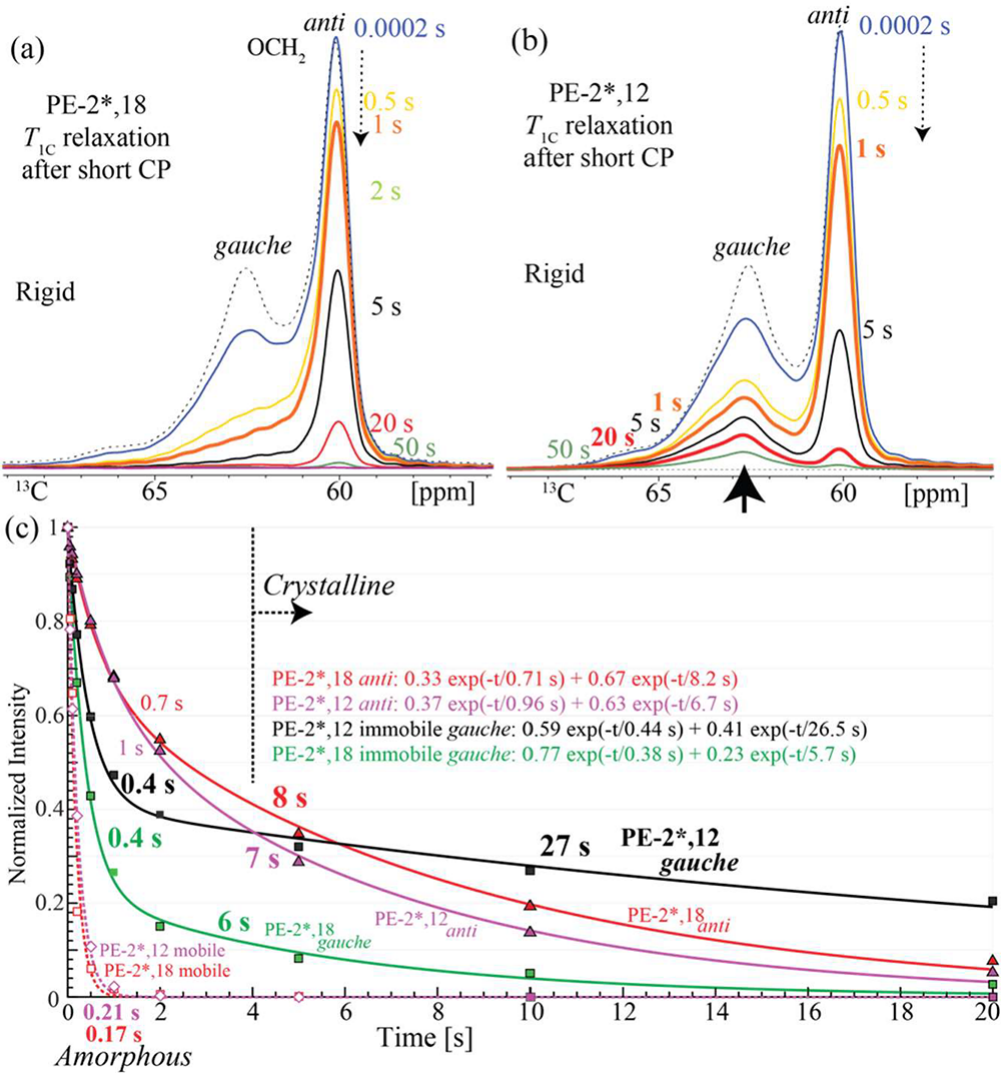
*T*_1C_ relaxation of rigid components in (a) PE-2*,18 and (b) PE-2*,12 after short cross-polarization and inverse dipolar dephasing (see Scheme S1 for the pulse sequence and Figure S1 for regular dipolar dephasing). The upward-pointing black arrow marks the rigid *gauche* components with unusually slow *T*_1C_ relaxation. (c) ^13^C spin–lattice relaxation data from selective spectra, with fit curves. Open symbols (lower left): mobile *gauche* components from Figure S2b; dashed curves: single-exponential fit curves with 0.21 and 0.17 s *T*_1C_ values. Squares: immobile *gauche*; triangles: immobile *anti*, from the spectra in (a, b). Spin–lattice relaxation times (in seconds) determined from the biexponential fits shown are marked on the curves.

In PE-2*,12, the disappearance of the interfacial component after 1 s in Figure 2b reveals a quite distinctive, slowly relaxing *gauche* band. Its relaxation is so unusually slow, see the black trace in Figure 2c with *T*_1C_ = 27 s, that this component becomes dominant even relative to the crystalline *anti* peak after ≥ 10 s of relaxation filtering. Figures 3 and 4 confirm the presence of this immobile (fully dipolar-dephasing) *gauche* component with unusually long *T*_1C_ relaxation time in slowly crystallized PE-2*,12 and in unlabeled PE-2,12, respectively. A sample made by ultraslow crystallization (at 0.1 K/min) was included here in order to make sure that these immobilized *gauche* segments were not kinetically trapped. Based on its slow relaxation, a small fraction of the slowly relaxing *gauche* component can be made visible even for PE-2*,18, see Figure 3b, after a 50-s *T*_1C_ filter.

**Figure 3:**
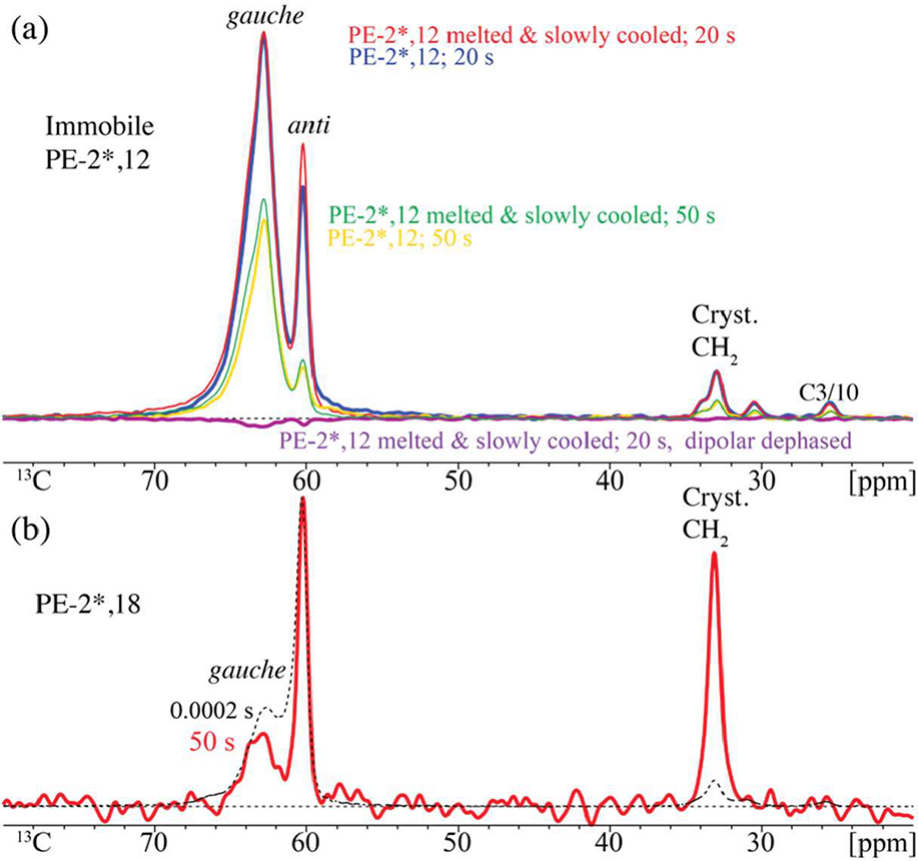
^13^C NMR spectra documenting immobilized (crystalline) *gauche* diol units in (a) two samples of PE-2*,12: regular treated and after ultraslow (0.1 K/min) recrystallization from the melt and (b) PE-2*,18. Spectra were obtained after short CP and the indicated long *T*_1C_ filter time ≥20 s. The magenta trace at the bottom in panel (a) was obtained after dipolar dephasing, showing no residual signal of mobile components. The red spectrum in panel (b) has been strongly scaled up to facilitate comparison of line shapes.

**Figure 4:**
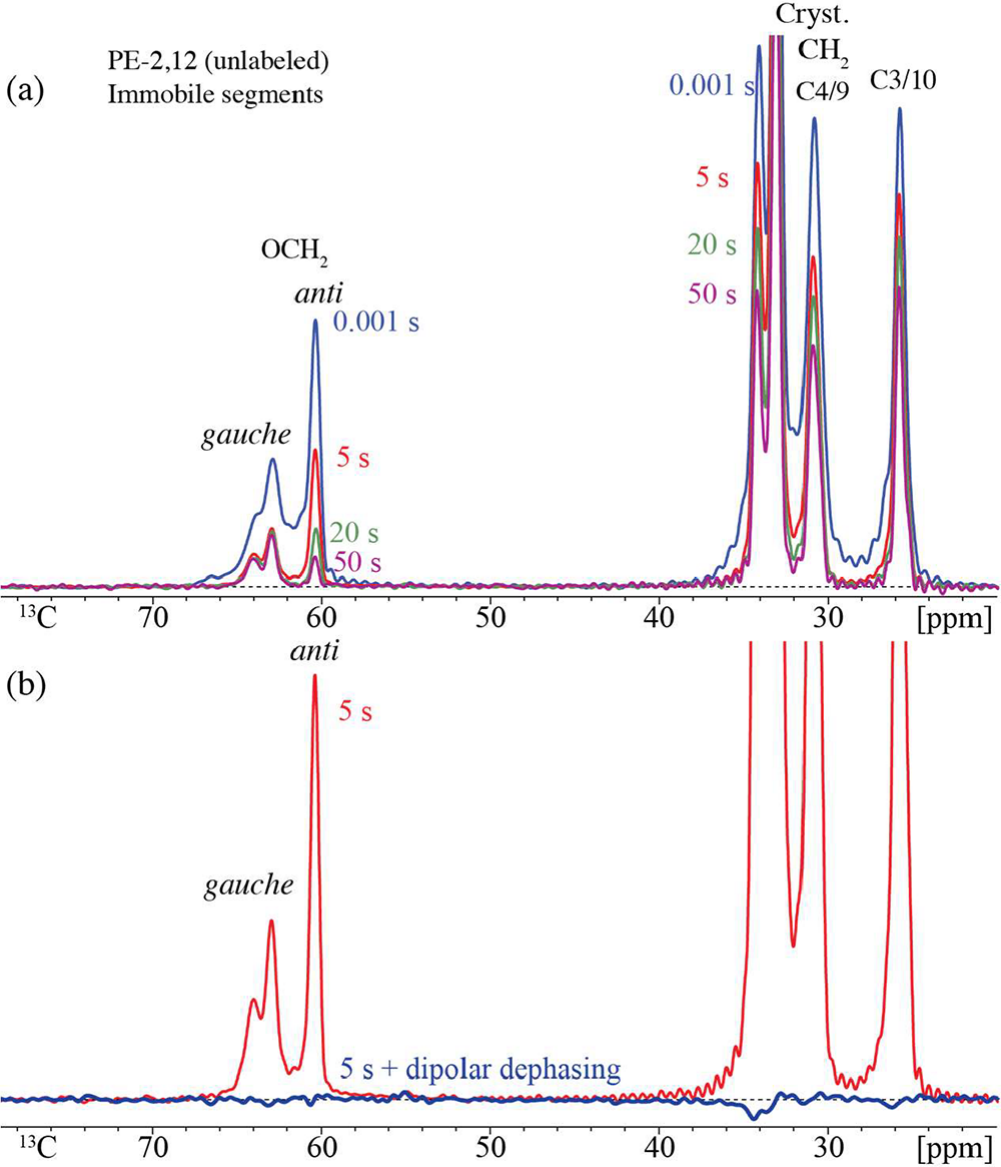
Short (0.1 ms) cross-polarization ^13^C NMR of crystalline segments in PE-2,12 in natural abundance (a) after *T*_1C_ filtering as indicated, up to 50 s, and (b) after a 5-s *T*_1C_ filter, without (thin red trace) and with (thick blue line) dipolar dephasing. The long *T*_1C_ and observed complete dipolar dephasing document that all segments detected here are rigid and crystalline, including the *gauche* OCH_2_ segments resonating near 63 and 64 ppm. Spectra were recorded at 6 kHz MAS.

Data from directly analogous relaxation experiments applied to immobile components in PE-2*,48 are shown in Figure 5. They document fast *T*_1C_ relaxation of the immobilized *gauche* segments resonating near 62.5 ppm, 2 orders of magnitude faster than that of the crystalline (CH_2_)_*n*_ peak at 33 ppm. After 5 s, no significant OCH_2_ signal remains, indicating that neither *gauche* nor *anti* segments are present in the crystallites. Similar experiments with direct polarization show that the fraction of highly mobile OCH_2_ segments is small, see Figure S3.

**Figure 5:**
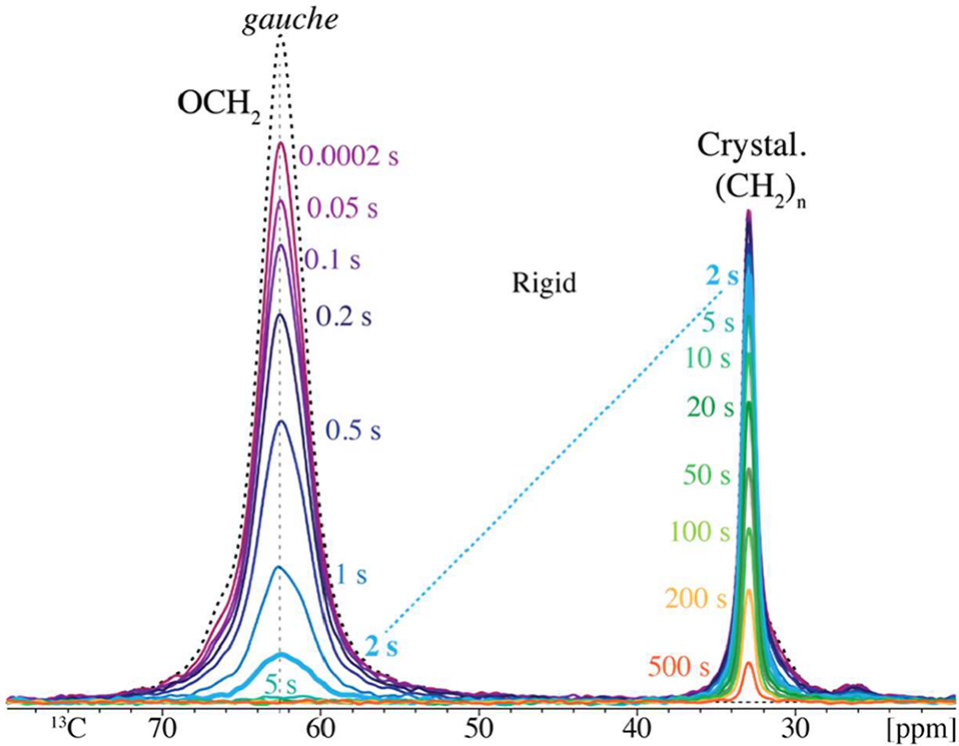
Spectrally resolved *T*_1C_ relaxation of components of limited-amplitude mobility in PE-2*,48, after selection by short CP and inverse dipolar dephasing. The dashed black curve at the top shows the full short-CP spectrum without inverse dipolar dephasing. The dashed line highlights the different relaxation of the two peaks after 2 s. See also Figure S3.

### ^1^H Spin Diffusion with ^13^C Detection

^1^H spin diffusion with ^13^C detection can conveniently provide information on domain proximities and with careful analysis,^24^ domain sizes up to 200 nm can be quantified. A simple *T*_2H_ filter was applied here to select the long-*T*_2H_^1^H magnetization of the highly mobile amorphous layers while suppressing the short-*T*_2H_ magnetization in the crystallites. This generates a steep gradient across the interface, which is gradually flattened by ^1^H spin diffusion out of the mobile into the rigid layers. The spatial diffusion process is accompanied by transfer of magnetization out of the 62.5-ppm, mobile *gauche*, into the 60.2 ppm, crystalline *anti*, OCH_2_ peak, see Figure 6a. The time course of that transfer contains information on the layer thicknesses, as re-equilibration occurs faster in thin layers than in thick ones.

**Figure 6:**
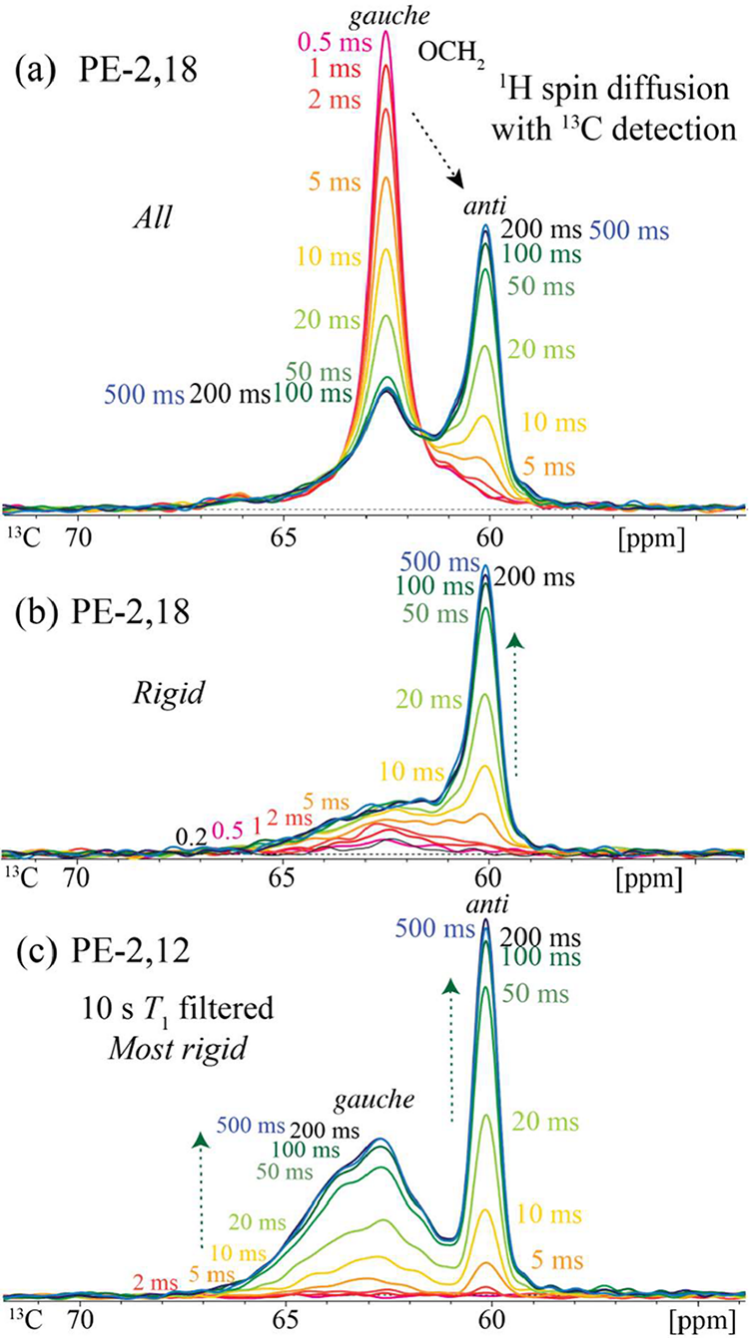
^1^H spin diffusion with ^13^C detection following a 150 μs *T*_2H_ filter in (a, b) PE-2*,18, after a 1-s *T*_1C_ filter and inverse dipolar dephasing in (b); and (c) PE-2*,12, after a 10-s *T*_1C_ filter.

Most interestingly, the location of the immobilized *gauche* components relative to the amorphous layers can be assessed in spectra detecting these immobilized components selectively, which can be achieved by a ^13^C–^1^H coupling based, double rigid filter combined with a sufficiently long *T*_1C_ filter, which strongly suppresses the mobile-*gauche* signal. The quality of the *gauche* suppression can be assessed by the absence of the narrow *gauche* peak from the spectra in Figure 6b. The series of spectra in Figure 6a,b show that in PE-2*,18, immobilized *gauche* OCH_2_ is reached by magnetization within ∼ 10 ms, long before slowly relaxing crystalline *anti*. This demonstrates that these immobilized *gauche* units are interfacial. In PE-2*,12, slowly relaxing *anti* and *gauche* OCH_2_ selected by a long *T*_1C_ filter are reached equally slowly by the magnetization diffusing out of the amorphous layers, see Figure 6c. This confirms that in PE-2*,12 both slowly relaxing *anti* and *gauche* OCH_2_ are distributed throughout the crystalline lamellae.

### CODEX ^13^C NMR: *Anti* vs *Gauche*

Centerband-only detection of exchange (CODEX) can quantitatively record changes in the anisotropic chemical shift due to motion or dipolar spin exchange on the 10^–4^–100 s time scale, with the high spectral resolution of magic-angle spinning.^25^ In this work, the focus is on dipolar ^13^C spin exchange, i.e., magnetization transferring from one ^13^C to a neighboring ^13^C based on their dipolar coupling (while the magnetization is longitudinal). This process is facilitated by ^13^C enrichment; for instance, spin exchange between the directly bonded ^13^C spins in O^13^CH_2_–^13^CH_2_O units occurs within a few milliseconds. Measurements on the corresponding material containing ^13^C only in natural abundance can be used to exclude frequency changes due to potential slow rotations of segments.

Most importantly for this study, CODEX can easily tell us whether the instantaneous (anisotropic) ^13^C chemical shifts of the directly bonded O^13^CH_2_ segments are nearly the same (*anti*) or distinctly different (*gauche*). The data in Figure 7 show that the immobile segments with *gauche* chemical shifts undergo spin exchange on the 3 ms time scale and (within 30 ms) reach the asymptotic level of 1/2 expected for exchange among the two O^13^CH_2_ sites. The *gauche* segments in PE-2,12 without isotopic labeling (open symbols at 50 and 100 ms near the top of Figure 7) do not show this drop, confirming that it is due to dipolar exchange and not segmental motion.

**Figure 7:**
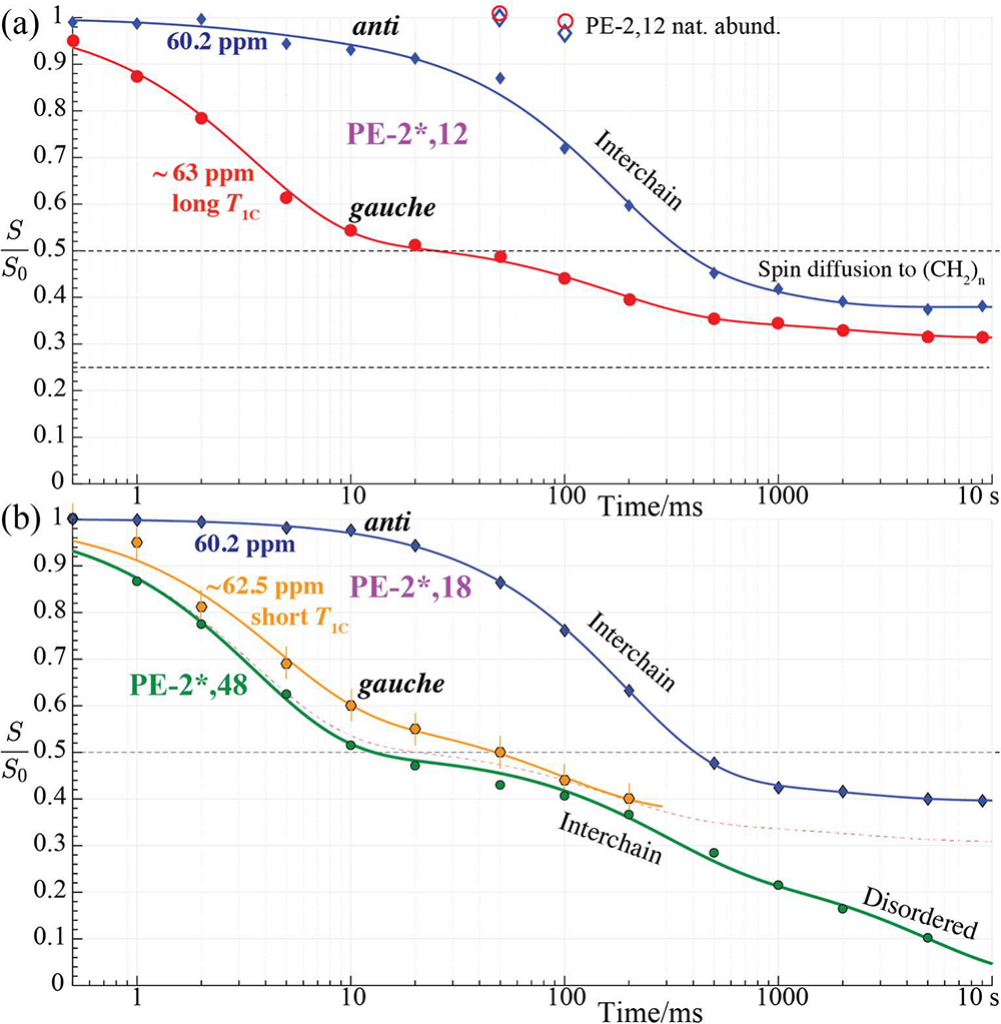
Normalized CODEX ^13^C NMR peak intensity of immobile OCH_2_ groups after 0.1 ms CP as a function of (logarithmic) mixing time. (a) PE-2*,12 (filled data points; diamonds: 60.2 ppm peak; circles: ∼63 ppm after 5-s *T*_1C_ filter) and PE-2,12 (natural abundance; open data points, after a 5 s *T*_1C_ filter). (b) PE-2*,18, showing crystalline *anti* at 60.2 ppm (after a 1-s *T*_1C_ filter; blue diamonds) and interfacial, partially mobile *gauche* at ∼62.5 ppm (after a 0.2 s *T*_1C_ filter; orange/black hexagons) and PE-2*,48 (green circles and fit curve, with the fit curve for rigid PE-2*,12 *gauche* segments from (a) repeated as a dashed red curve for reference). Error bars are smaller than the symbol size, except where shown.

At longer times (note the logarithmic time axis in Figure 7), ^13^C spin exchange occurs between neighboring ^13^C-labeled chains and CODEX provides information about chain packing because the ^13^C frequencies are generally different in chains whose zigzag planes are not parallel, as discussed below.

## Discussion

The following discussion focuses on *gauche* vs *anti* diol units in the amorphous, interfacial and crystalline layers. Other, incidental observations, e.g., of (CH_2_)_*n*_ signals or chain ends, are discussed in the SI.

### NMR Characteristics of OCH_2_ Motion

The spectra of PE-2*,12 and PE-2*,18 in Figure 1 each exhibit two narrow peaks, at 60.2 and 62.8 ppm, assigned to crystalline *anti* and mobile *gauche* amorphous OCH_2_ units, respectively. This highlights that spectral lines in solid-state NMR can be sharp for two different reasons: (i) in a crystal, segments related by symmetry have the same environment and therefore the same chemical shift; and (ii) fast dynamic averaging over different conformations and packing environments, with large motional amplitude, results in a single average chemical shift. This motional averaging is the mechanism that produces sharp lines in solution NMR.

Crystalline versus liquid-like sharp lines of CH_*n*_ sites can be reliably distinguished in ^13^C ssNMR based on their different relaxation behavior and average C–H dipolar coupling strengths. Fast large-amplitude motions of C–H bonds produce strongly fluctuating magnetic fields with rates near the Larmor frequency (the NMR frequency; 2π 100 MHz in our magnet), which drive relaxation of ^13^C with the spin–lattice relaxation time *T*_1C_. As a result, ^13^C magnetization of highly mobile segments relaxes fast, on the 0.2-s scale, while *T*_1C_ exceeds 5 s for more or less immobile crystalline sites. Data in Figure 2(c) document these characteristic *T*_1C_ differences. On this basis, the signals of the mobile segments can be observed selectively in direct-polarization NMR with ∼1-s recycle delay or suppressed by a ≥1-s *T*_1C_ filter. Because fast large-amplitude motions average the ^13^C–^1^H dipolar couplings, they can also be documented or excluded by dipolar dephasing, i.e., switching off of ^1^H decoupling for 68 μs. In rigid CH_2_ and CH groups, dephasing is complete (0% remaining), while peaks of liquids do not dephase (100% remaining). All these characteristics are compiled in the first two rows of Table 1.

**Table 1:** ^13^C NMR Characteristics of Major OCH_2_ Signals in the Polyesters Studied Here

chemical shift (ppm)	line width	conformation	dipolar dephasing to (%)	*T*_1C_ (s)	mobility	disorder	location^d^
60.2	sharp	*anti* ^a^	0	8	rigid	low	crystal
62.8	sharp	*gauche* ^b^	70–90	0.2	highly mobile	high, dynamic	amorphous
62.5	broad	*gauche* ^a^	∼15	∼0.5	limited	static	interface
∼63	^c^	*gauche* ^a^	0	27	rigid	low	crystal

a From chemical shift and CODEX NMR.

b From chemical shift.

c Broad with ^13^C enrichment; sharp lines at 63 and 64 ppm with ^13^C in natural abundance.

d Confirmed by ^1^H spin diffusion with ^13^C detection.

Inhomogeneously broadened ^13^C NMR lines (under ^1^H decoupling), as observed near 62.5 ppm in Figure S4c, are the hallmark of immobilized disordered segments. The combination of limited mobility and disorder is expected at the crystal–amorphous interface. Such segments often undergo fast motions of limited amplitude, due to loose packing. If the rate of motion is near the Larmor frequency, *T*_1C_ can still be fairly short (∼0.5 s) while the averaging of the C–H dipolar coupling is limited and therefore significant ^13^C­{^1^H} dipolar dephasing, to ≤20%, is observed, as summarized in the third row of Table 1.

Finally, homogeneous ^13^C NMR line-broadening can arise in ^13^C_2_ spin pairs when the two ^13^C spins have different instantaneous frequencies, as is the case for immobile *gauche* segments. Since the chemical-shift difference and the ^13^C–^13^C dipolar coupling do not commute with each other and do not commute at different rotor orientations, the “homogeneous” Hamiltonian^26^ results in line broadening that is removed only at very high spinning frequencies; it is avoided in samples without ^13^C labeling.

### *Gauche* vs *Anti* OCH_2_

The OCH_2_ spectrum of PE-2*,12 contains at least five distinct components (mobile amorphous *gauche*, immobile interfacial *gauche*, crystalline *gauche*, crystalline *anti*, and a minor mobile *anti* component tentatively assigned as unlayered crystalline or as interfacial). The assignment of *gauche* conformations is central to this work and therefore needs to be justified convincingly. The most reliable assignment to *gauche* and *anti* diol units in the pairwise ^13^C-labeled samples studied here comes from CODEX ^13^C NMR while also considering dipolar dephasing: A component with pronounced dipolar dephasing to <20% and with less than 20% CODEX dephasing within 100 ms is *anti* (and immobile): both OCH_2_ groups are relatively immobile and have nearly the same instantaneous, anisotropic chemical shift. On this basis, the right-most, sharp peak in the OCH_2_ spectra of PE-2*,12 and PE-2*,18, near 60.2 ppm, is assigned to immobile *anti* units.

The sharp left OCH_2_ peak, near 62.8 ppm, is from highly mobile segments, according to its slow cross-polarization, incomplete dipolar dephasing and fast spin–lattice relaxation (*T*_1C_ = 0.2 s). It can be assigned to OCH_2_ groups in the amorphous layers.

Underlying the sharp peak near 62.8 ppm are broader signals from immobilized components with fast CP, significant dipolar dephasing, and moderate to long *T*_1C_ selectively observed in Figures 2b, 3, 4, and 6; these are identified as *gauche* by fast two-site dipolar exchange, on the 10 ms scale, observed by CODEX NMR, see Figure 7. While this exchange could in principle also arise from diffusive motions (but not chain flips,^27^ which have little effect on the ^13^C chemical-shift anisotropy), it would be highly unlikely to occur exactly on the 2 ms time scale of the dipolar exchange and it would not plateau near 0.5. This improbable scenario is rigorously ruled out by CODEX experiments on the immobile *gauche* peak in the corresponding unlabeled material, where only motional but no one-bond dipolar exchange can occur on the 100 ms time scale. If there is less than 10% exchange within 100 ms, the drop to 50% in the labeled material must be dipolar in nature, from a *gauche* OCH_2_–CH_2_O segment. This is the case for PE-2,12, see the open data points at 50 and 100 ms in the top center of Figure 7a.

These observations allow us to conclude that the OCH_2_ peaks near 62.5 ppm are from *gauche* diol units, while the peak near 60 ppm is from *anti* units. This is in excellent agreement with the published^19,23^ spectral observations for the amorphous *gauche* and crystalline *anti* ester OCH_2_ groups in PET.

### Highly Mobile *Gauche* OCH_2_

An OCH_2_ component with *gauche* chemical shift, small ^13^C line width, weak C–H dipolar couplings, and extremely short *T*_1C_ = 0.2 s is easily detected after direct polarization with ≥1 s recycle delay and dipolar dephasing, see Figures 1b, 6a, and S6a. It can clearly be assigned to the soft amorphous layers with dynamic disorder, whose softening temperatures *T*_g_ are below 0 °C.^28^ While we describe this component simply as highly mobile *gauche*, it is likely a dynamic average of 70–90% *gauche* and 10–30% *anti*, similar to the frozen-in distribution in PET.^19^ Mobile OCH_2_ is quite insignificant in PE-2*,48, where most OCH_2_ is interfacial, see the broad band in Figure 1b.

### Crystalline *Gauche* OCH_2_

Given that PE and the (CH_2_)_*n*_ segments in the polyesters studied here take an all-*anti* planar zigzag conformation in their crystallites, the most surprising finding in this study is crystalline *gauche* OCH_2_–CH_2_O segments of considerable concentration in PE-2,12 and at a low level in PE-2,18. The *gauche* conformation is indicated by the ^13^C chemical shift >62 ppm and proven by a fast (∼10 ms) decay to 50% in CODEX ^13^C NMR. The crystalline character of these *gauche* segments is underlined by their unusually long *T*_1C_, four times longer even than for crystalline *anti* OCH_2_–CH_2_O, see Figure 2c. It is further supported by the sharpness of the peak at 63 ppm in Figure 4b, in the spectrum of the natural-abundance sample (without the ^13^C–^13^C dipolar broadening that occurs in PE-2*,12 because the instantaneous chemical shifts of the coupled ^13^C nuclei are different). The location of the *gauche* segments inside the crystal has been proven by slow ^1^H spin diffusion from the mobile amorphous layers. After a *T*_1C_ filter of 50 s duration, in the spectrum of PE-2*,12, the crystalline *gauche* is by far the dominant signal, see Figure 3a. Without crystalline *gauche*, the apparent crystallinity from the *anti* OCH_2_ signal area fraction would be only ∼35%, far lower than the value of 62 ± 4% from analysis of wide-angle X-ray diffraction patterns and quantitative ^13^C NMR spectra (see Table S1 and Figures S6–S8). *Gauche* conformations at relatively high OCH_2_–CH_2_O concentrations (PE-2,12 having the highest oxygen concentration among the samples studied here) are consistent with the known intrinsic preference of the OCH_2_–CH_2_O torsion for *gauche* conformations.^19^

Wondering if the crystalline *gauche* conformers were kinetically trapped, unstable species, we melted an aliquot of PE-2*,12 and kept it at 100 °C for 20 min to erase any thermal history, then cooled it from 75 to 50 °C at 0.1 °C/min for nearly isothermal crystallization. This processing actually decreased the crystalline *anti* peak, see Figure S4, while the crystalline *gauche* peak did not go down. Overall, the crystalline *gauche* has been detected in three different samples (original PE-2*,12, recrystallized PE-2*,12, and unlabeled PE-2,12) and must therefore be considered a stable feature of this material. Notably, a *gauche* conformation is adopted by the diol units in the amorphous regions and at the amorphous–crystalline interfaces, i.e., whenever they are not constrained by crystal packing. This is strong evidence that *gauche* diol units are energetically favorable, which can result in their appearance even in the crystal.

The crystalline *gauche* diol in PE-2,12 seems to be roughly in a 1:1 ratio with the interfacial *gauche* segments, according to the *T*_1C_ decay series, while it is in a 1:2 ratio to the crystalline *anti* units. Thus crystalline *anti* and *gauche* combined add to about 50% of all OCH_2_ in PE-2*,12, comparable to the crystalline *anti* OCH_2_ in PE-2*,18. Why both fractions combined still fall short of the crystallinity of ≥57% (see Table S1) is explained below.

While ^1^H spin diffusion has shown that crystalline *gauche* and *anti* diols are distributed similarly throughout the crystals of PE-2*,12, it does not answer the questions if there are randomly mixed layers of *gauche* and *anti* diols. Spin–lattice relaxation indicates that there are not. *T*_1C_ is much longer for the *gauche* than the *anti* segments, on the scale of tens of seconds; this difference in *T*_1C_ proves clustering of crystalline *gauche* segments: ^13^C spin exchange between neighboring chains occurs already on the 2-s time scale, as documented by CODEX, and would equalize *T*_1C_ in randomly mixed layers of *gauche* and *anti* segments.

At a low level, PE-2*,18 also appears to contain crystalline *gauche* segments. Due to their unusually long *T*_1C_ relaxation time, they can be separated from all overlapping signals by a 20-s *T*_1C_ filter, see Figure 3b. However, their low concentration is difficult to estimate due to the distortion of the spectral intensities by the long relaxation-time filter.

The detection of *gauche* conformers in the crystallites of PE-2,12 is very unexpected in the context of polyethylene, since chains in the crystallites of PE are all-*anti* while *gauche* conformers have traditionally been associated with the noncrystalline layers, or with defects.^20,21^ Nevertheless, *gauche* OCH_2_–CH_2_O conformers can be part of extended chain structures; to demonstrate this, Figure 8 shows examples. The observation of two crystalline *gauche* peaks in Figure 4b, whose peak areas are similar, can be interpreted as arising from the two OCH_2_ sites in a given *gauche* conformer that is flanked asymmetrically by different O–C torsion angles, as in the model in Figure 8b. While the widths of the *gauche* peaks at 63 and 64 ppm in Figure 4 differ by a factor of ∼1.7, their indistinguishably long *T*_1C_, much different from the *anti* segments, see Figure 4a, suggests a similar environment of the 63- and 64-ppm of OCH_2_ sites. That the 63- and 64-ppm crystalline OCH_2_ groups are directly bonded is supported by a line-shape change in 2D exchange spectra within a mixing of 200 ms, see Figure S9, which can be best interpreted in terms of one-bond dipolar spin exchange between the two *gauche* peaks at 63 and 64 ppm.

**Figure 8:**
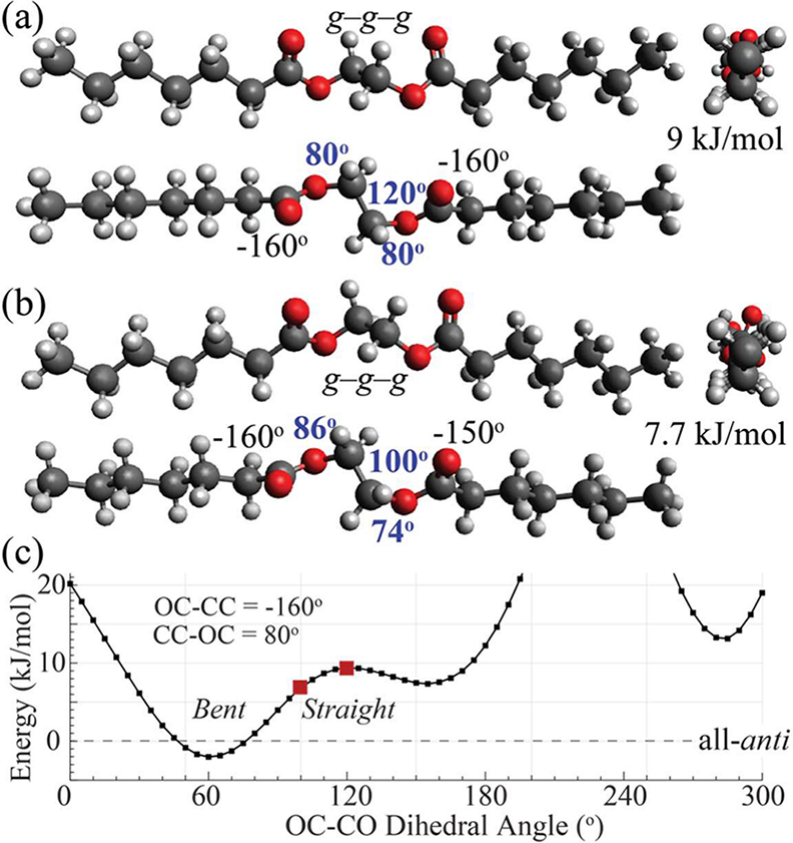
Models of extended ethylene glycol diester chains with central OCH_2_–CH_2_O segments far from *anti*. The adjacent CC-OC torsions are both *gauche* near +80°. (a) Three views of a model with an OC–CO torsion angle of 120° and adjacent CC-OC torsions of +80°, which produces a straight chain that is ∼9 kJ/mol less stable than the all-*anti* chain; (b) views of a model with an OC–CO torsion angle of 100°, in an only slightly bent chain that is ∼7.7 kJ/mol above all-*anti*. The adjacent CC-OC torsions are *gauche* at +74° and +86°, which produces an OCH_2_ isotropic shift difference of 1.5 ppm, large enough to match the experiment, see Figure 4. (c) Energy of a chain segment as a function of the OC–CO torsion angle; note the deep minimum showing that bent chains with *gauche–gauche–gauche* diols can be more stable than the all-*anti* chain; however, because they are not straight enough, they can only exist at the crystal surface or in the amorphous layers. It seems likely that to reduce energy, the OC–CO torsion in PE-2,12 is closer to the minimum, e.g., taking a value of 80°, and the bend in the chain is accommodated by other structural adjustments.

### Immobilized Interfacial *Gauche* OCH_2_

All three samples show an OCH_2_ component with *gauche* chemical shift, significant ^13^C line width, strong C–H dipolar couplings (fast CP and pronounced dipolar dephasing), and fairly short *T*_1C_ ≤ 0.5 s. Together these observations indicate fast motions of limited amplitude in a disordered environment. In PE-2*,48, this component is most apparent because it is the main form of OCH_2_ present, and CODEX has clearly proved that it is *gauche*. The limited decrease in the CODEX intensities in Figure 7b also implies the absence of large-amplitude motions on the time scale between 10 ms and 1 s. In PE-2*,18, the CODEX data of the interfacial *gauche* component in Figure 7b appear to show a slightly slower initial decay not quite to 0.5, which is consistent with some motional averaging of the OCH_2_ chemical-shift anisotropy of these interfacial segments in proximity to highly mobile segments in the amorphous layer. Fast spin diffusion from the amorphous layers, see Figure 6b, proves their interfacial locations.

### Crystallinity vs OCH_2_–CH_2_O *Anti* Fraction

In none of the three materials studied here does the OCH_2_–CH_2_O *anti* signal fraction with long *T*_1C_ reach a value corresponding to the crystallinity as listed in Table S1 (57–76%), determined by NMR and X-ray peak-area analyses (see the SI, Figures S6–S8). In the most extreme case, in PE-2*,48, the intensity at the *anti* chemical shift of ∼60 ppm is only a moderate shoulder (≤20%) and the associated *T*_1C_ is <1 s, see Figure 5; our model with OCH_2_ groups mostly at the crystal–amorphous interfaces accounts for this observation. This example of PE-2*,48 highlights that the OCH_2_ crystallinity does not have to match the (CH_2_)_*n*_ crystallinity, which is also true, in a more subtle form, in PE-2,18 and PE-2,12 as discussed here. Integration of the 60-ppm peaks in Figure 1 shows that in PE-2*,12 and PE-2*,18, the *anti* OCH_2_ fraction is only ∼33 and ∼45%, respectively, clearly below the crystallinity of ≥57%, see Table S1.

In PE-2*,12, this strong discrepancy is reduced when the intensity of the immobile, slow-relaxing, crystalline *gauche* OCH_2_ is added to that of the crystalline *anti* OCH_2_ peak. The remaining discrepancy is removed through the effect of interfacial layers as discussed for PE-2,18 in the following.

The crystalline *anti* OCH_2_ peak in PE-2*,18 is relatively larger than in PE-2*,12 (see Figure 1), but by itself is still too small (≤45%) to account for the crystallinity of ≥57% (see Table S1). The interfacial *gauche* OCH_2_ can resolve this discrepancy, e.g., in a model with two *anti* diol layers (i.e., three (CH_2_)_16_ layers) in the crystal and two interfacial *gauche* OCH_2_ layers, see Figure 9: with only two crystalline *anti* OCH_2_ layers per three crystalline (CH_2_)_16_ layers, the *anti* OCH_2_ crystallinity is only 2/3 of the regular (CH_2_)_16_ crystallinity.

**Figure 9:**
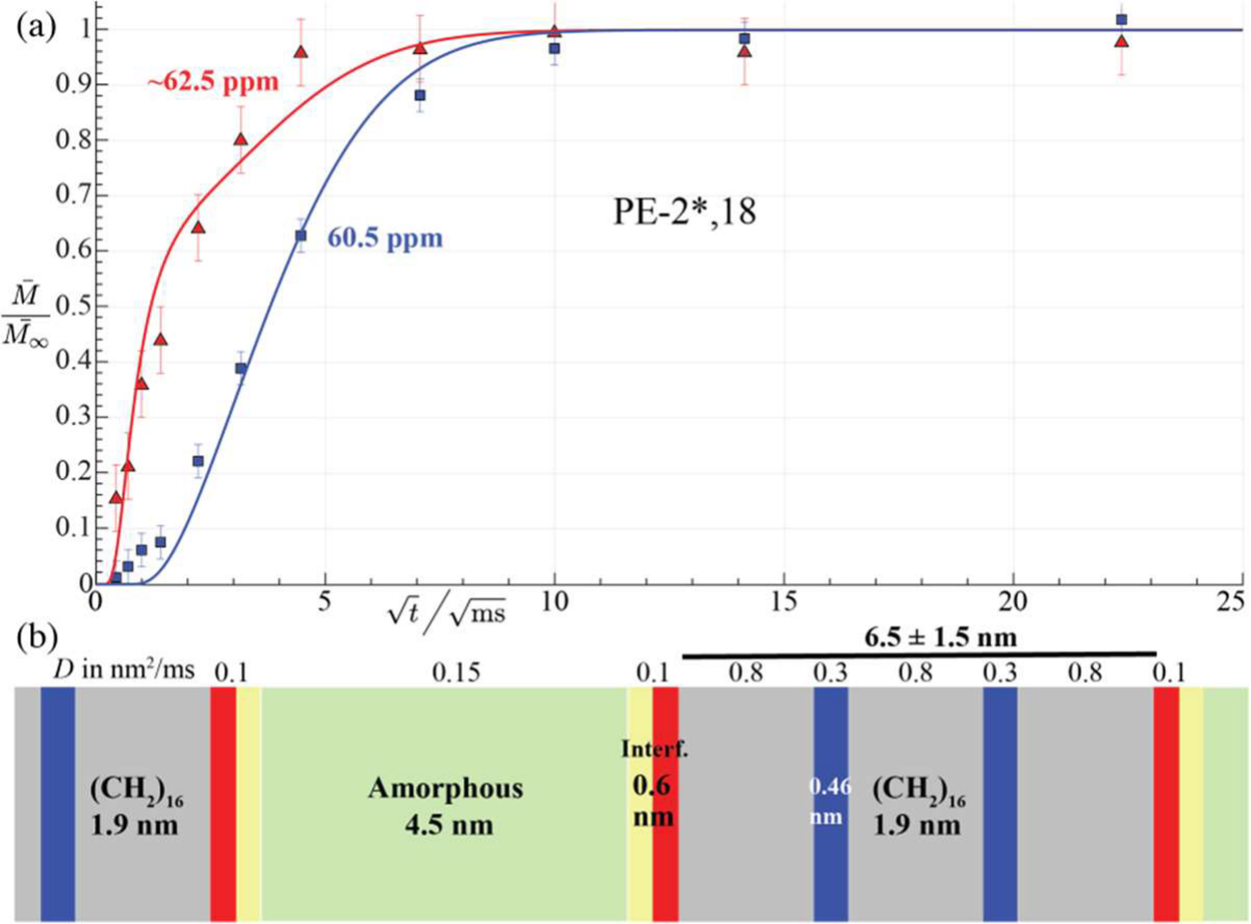
Supramolecular structure of PE-2*,18. (a) Normalized intensities from the ^13^C-detected ^1^H spin diffusion experiments shown in Figure 6b. Red triangles and trace: rigid interfacial *gauche* O^13^CH_2_; blue squares and curve: rigid *anti* O^13^CH_2_. (b) Layered model used to produce the fit curves in (a). Diol/diester layers at the interface (with *gauche* OCH_2_–CH_2_O) are shown in red and those in the crystallites in blue (with *anti* OCH_2_–CH_2_O). The thin yellow layers consist of moderately mobile (CH_2_)_*n*_ and COO groups, which are neither magnetization sources nor detected here.

### Chain Packing from Long-Time CODEX

CODEX normalized-intensity *S*/*S*_0_ data were measured over more than 4 orders of magnitude in time, from 0.5 ms to 10 s, see Figure 7. On the shortest scale from 0.5 to 100 ms, CODEX probes the relative orientation of directly bonded ^13^C spins, while between 100 and 2000 ms, interchain spin exchange can be detected. The observed drop of the *anti* intensity on this longer time scale can be attributed to spin exchange between the two different zigzag chain orientations in the herringbone crystal packing characteristic of orthorhombic polyethylene and the polyesters studied here.^8,10^ At longer times, exchange occurs not only between the O^13^CH_2_ groups of different chains but also with ^13^C in natural abundance in the diester segments, resulting in an asymptote sloping to below 50%.

The interfacial *gauche* segments in PE-2*,48 also appear to show this 2-fold decrease due to interchain exchange, but at longer times more exchange is observed, resulting in a further drop to <1/8. This is an indication of packing disorder with many differently oriented nearby O^13^CH_2_ segments, which is reasonable for the disordered interfacial layers in this material with their inhomogeneous signal broadening indicating a range of packing environments.

The crystalline *gauche* segments in PE-2*,12, beyond their initial intensity drop to 50%, show unexpectedly little further exchange. This might indicate that there are few ^13^C spins in the vicinity (no ester layering) or that neighboring *gauche* segments are parallel, with OCH_2_ groups of the same instantaneous chemical shift.

### Layered Models

The detailed information from NMR spectroscopy and ^1^H spin diffusion, combined with domain sizes estimated by SAXS analysis^8,10^ and mid-angle X-ray scattering (MAXS) showing a reflection from regularly spaced layers of esters in PE-2,12^28^ and PE-2,18,^16,8,10^ can be used to construct realistic models of the layered supramolecular structure in a typical semicrystalline repeat unit of each of the three materials studied. For instance, NMR of PE-2*,48 shows a PE-like pure (CH_2_)_*n*_ crystal without crystalline esters while the OCH_2_ units are immobilized and disordered, clearly pointing to ester layers at every crystal–amorphous interface. Thus, layered models are well supported by the data and we used them in our analysis of ^1^H spin diffusion and crystallinity.

Figure 9a presents intensity data from the series of spectra of PE-2*,18 in Figure 6b as a function of the square-root of the ^1^H spin diffusion time. The signal of the immobilized disordered *gauche* OCH_2_ component rises faster than that of the crystalline *anti* layers, showing that these *gauche* segments are interfacial, as seen in the model sketched in Figure 9b, which provided the quantitative fits (solid lines) to the data in Figure 9a. As discussed above, this model with two crystalline diester layers is also consistent with the signal-intensity data in Figure 1 and with the diffraction peak from the ester and ethylene glycol layers in MAXS,^16,8,10^ see the simulations^29^ in the SI and Figure S5.

Figure 10a provides a similar plot of spin diffusion data of the most rigid OCH_2_ components in PE-2*,12 (from the series of spectra in Figure 6c). The signals of the ordered, rigid *gauche* and *anti* components rise fairly slowly together, showing that both, including the long-*T*_1C_ *gauche* units, are dispersed (in layers) throughout the crystallites. A distinct MAXS peak of PE-2,12^28^ confirms that its diols are layered, and simulation for the model in Figure 10b provides a good match with the observed^28^ MAXS peak, see Figure S5g. It is currently not known if crystalline *gauche* alternate with crystalline *anti* OCH_2_ layers.

**Figure 10:**
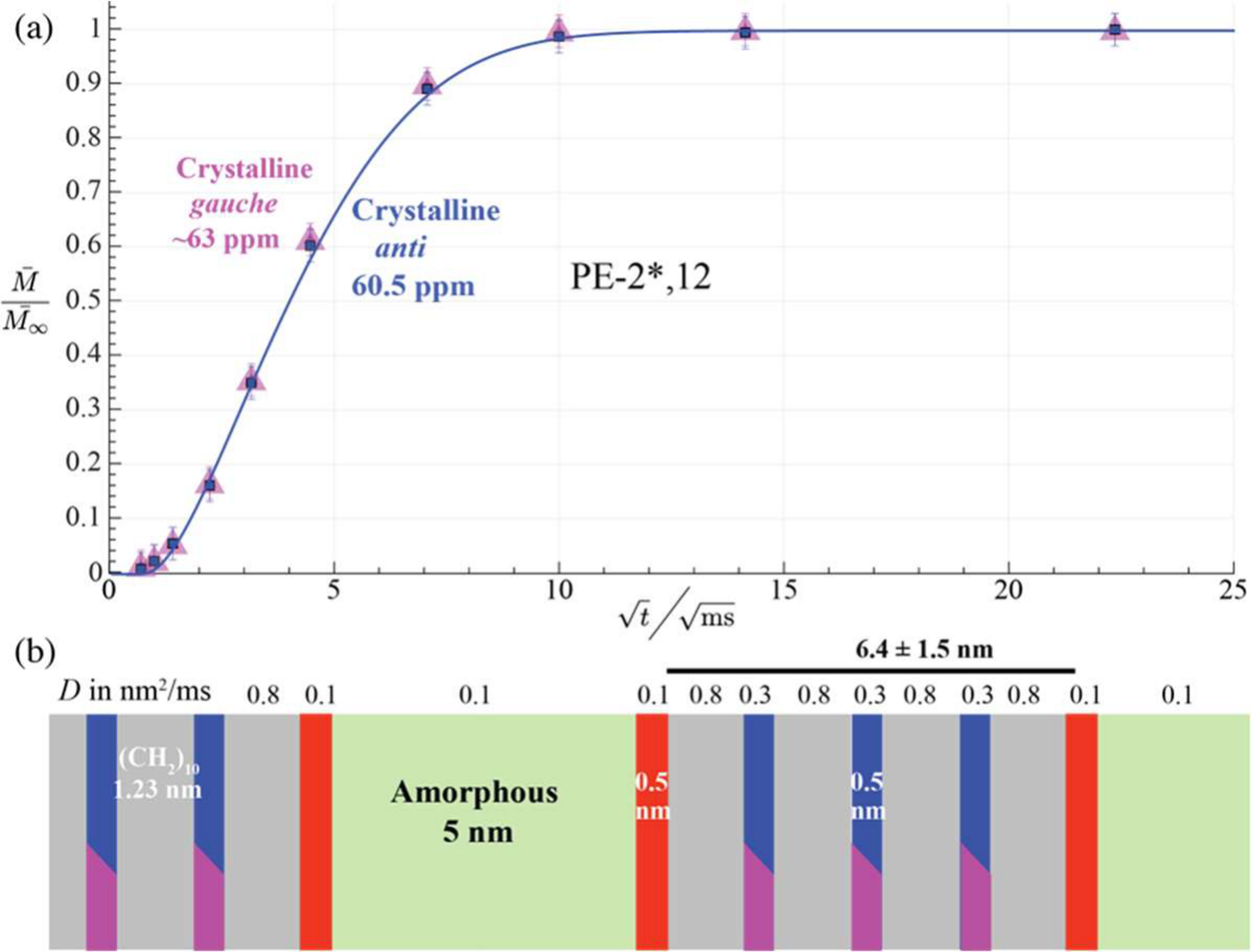
Supramolecular structure of PE-2*,12. (a) Normalized intensities from the ^13^C-detected ^1^H spin diffusion experiments shown in Figure 6c. Magenta/purple triangles: rigid *gauche* O^13^CH_2_; blue squares: rigid crystalline *anti* O^13^CH_2_. (b) Layered model used to produce the fit curve in (a). Diol/diester layers at the interfaces (with *gauche* OCH_2_–CH_2_O) are shown in red, those in the crystallites in blue (*anti* OCH_2_–CH_2_O) and magenta (rigid *gauche* OCH_2_–CH_2_O).

### Chemical Control of Morphology in PE-2,48

The semicrystalline supramolecular structure of PE-2*,48 is strongly constrained by the observed exclusion of OCH_2_ groups from the crystal interior. This implies chemical control, at high crystallinity (76%, see Table S1), of the crystallite thickness imposed by the regular spacing of the diester layers. Given that no OCH_2_ groups are found inside the crystals and most OCH_2_ groups are interfacial, the rows of 46 CH_2_ groups plus two ester carbons of the dicarboxylate form the crystallite. With 0.125 nm per CH_2_ group and a chain tilt of 35° relative to the crystal surface normal, the crystallite thickness is about 4.9 nm. The rise of the intensity of the (CH_2_)_*n*_ peak after ^1^H spin diffusion from the noncrystalline layers is fitted for such a model in Figure 11, with the thickness of the amorphous layers deduced from the crystallinity. These layers appear to consist mostly of disordered (CH_2_)_46_ segments connecting the interfacial diester layers.

**Figure 11:**
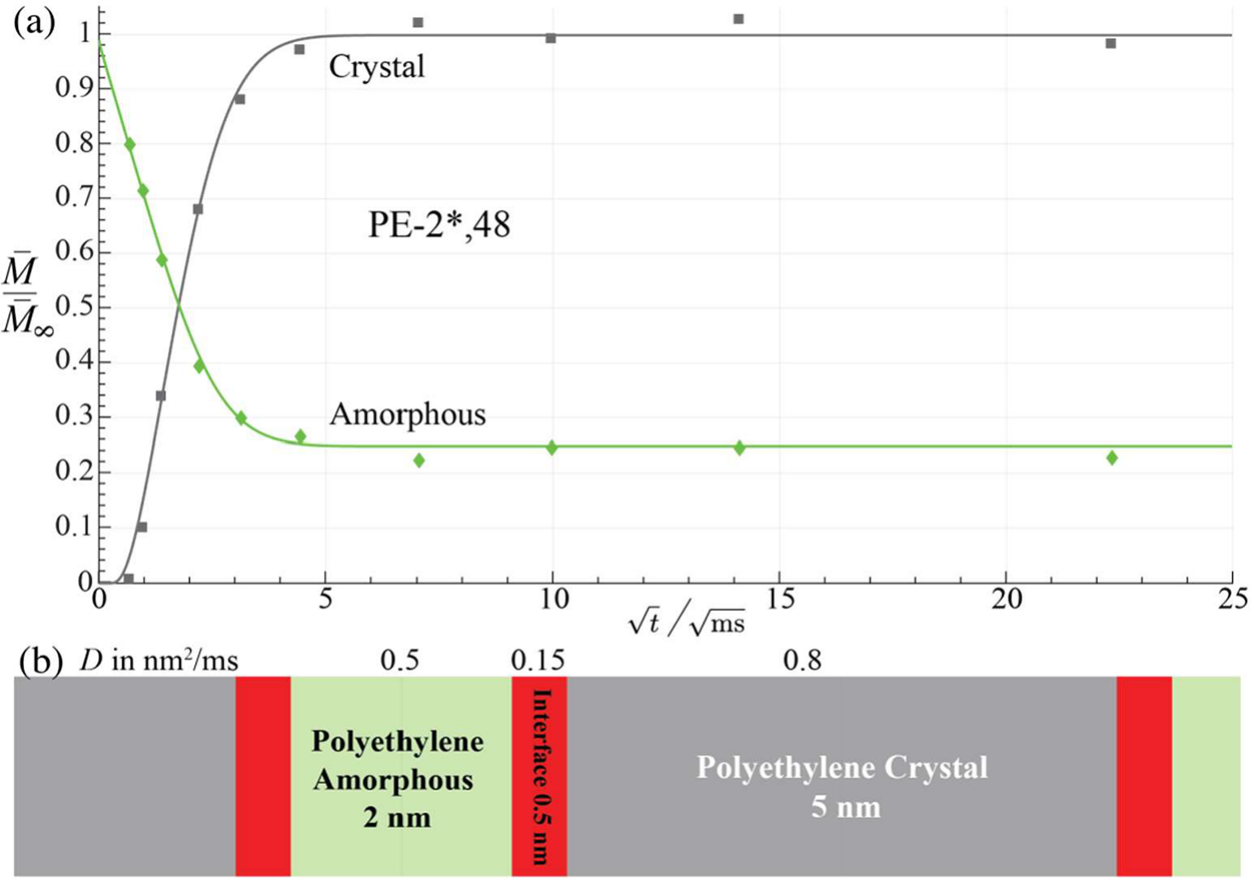
Supramolecular structure of PE-2*,48. (a) Normalized intensities from ^13^C-detected ^1^H spin diffusion after a 15 μs *T*_2H_ filter. Dark gray squares and trace: Crystalline (CH_2_)_*n*_ after a 5 s *T*_1C_ filter. Green diamonds: mobile *gauche* O^13^CH_2_ selected by dipolar dephasing. (b) Layered model used to produce the fit curves in (a). Diol/diester layers at the interfaces (with *gauche* OCH_2_–CH_2_O) are shown in red.

## Conclusions

The conformational and supramolecular structure of three polyethylene-like polyesters, with a focus on their ^13^C-labeled ethylene glycol segments, has been determined by advanced solid-state NMR with mobility-based filters, ^13^C dipolar exchange, and ^1^H spin diffusion with ^13^C detection. Immobilized *gauche* OCH_2_–CH_2_O segments are completely dominant in PE-2,48 while representing major components in PE-2,12 and PE-2,18. This is documented in various ^13^C NMR spectra, starting with dramatic differences between the three polyesters’ quantitative OCH_2_ spectral patterns. The samples contain immobilized interfacial *gauche* OCH_2_-CH_2_O segments proved by fast CODEX dephasing to 0.5. In PE-2*,48 these are the main type of OCH_2_ groups and clearly interfacial, being neither part of the *anti*-dominated crystalline layers nor located in the cores of the mobile amorphous layers; their broad lines, significant dipolar dephasing, and fast *T*_1C_ relaxation confirm this conclusion. In PE-2*,18, the interfacial location of the immobilized *gauche* OCH_2_ groups is proven by fast ^1^H spin diffusion from the mobile amorphous layers. In PE-2,12, signals of crystalline *gauche* OCH_2_ segments were separated from all others based on their unusually slow *T*_1C_ relaxation. The *gauche* conformation is indicated by their chemical shift and proven by CODEX spin exchange within 3 ms that is absent in natural abundance. Their location inside the crystalline layers, indicated by the characteristically long *T*_1C_, is proven by slow ^1^H spin diffusion from the amorphous layers, exactly tracking that of the crystalline *anti* segments. The large concentration of crystalline *gauche* segments, about 1/3 of the crystalline OCH_2_, in PE-2,12 has been confirmed in three different samples. PE-2,12 contains at least three distinct types of *gauche* OCH_2_–CH_2_O units: mobile amorphous with *T*_1C_ = 0.2 s, immobilized interfacial with *T*_1C_ ∼ 0.5 s, and crystalline with long *T*_1C_ ∼ 27 s, longer than for crystalline *anti* segments (*T*_1C_ = 7 s).

Our findings have strong general implications with regards to supramolecular structures of polyethylene-like aliphatic polyesters of varying methylene run-lengths. A highly crystalline polyester structure with an all-polyethylene crystal is observed for the first time, which is quite fundamental for understanding or predicting the degradability and recyclability of such ultralong chain-length polyethylene-like polyesters. Furthermore, the thickness of this polyethylene crystal was fixed by the run-length of methylene units, which in turn controls the resulting polyester thermal properties and implies tunability of lamellar thickness within a range of (ultra)­long-chain diacid segments. The exclusion of ester groups from the crystals is enforced by structural consequences of a short-chain functional group containing motif, particularly the prominence of *gauche* conformations of the C_2_-diol unit that favors ester and diol layers at the crystal surfaces. The conformation and location of these ester-containing motifs across amorphous, crystalline, and interfacial structural domains could have implications for the accessibility of the ester bonds to microbial extracellular enzymes, which is a prerequisite for their biodegradability by microorganisms. Elucidating relationships between polyester structure and biodegradability is the focus of future work. These aforementioned structural aspects go beyond all observations on ‘polyethylene-like’ long- and ultralong-chain-polyesters made so far, and were enabled by advanced solid-state NMR spectroscopic experiments enhanced by ^13^C-labeling of the C_2_-diol monomer units.

## Supplementary Material



## Notes

Experimental methods with an overview of NMR pulse programs used; table of key physicochemical properties of the samples; simulation of mid-angle X-ray scattering peaks; additional discussion of (CH_2_)_*n*_ signals, crystallinity, and OH chain ends; NMR spectra and WAXD patterns for crystallinity analysis; and additional quantitative or selective solid-state NMR spectra (PDF)

## References

[1] Zhu Y., Romain C., Williams C. K. (2016). Sustainable Polymers from Renewable
Resources. Nature.

[2] Coates G. W., Getzler Y. D. Y. L. (2020). Chemical Recycling
to Monomer for an Ideal, Circular
Polymer Economy. Nat, Rev. Mater..

[3] Vollmer I., Jenks M. J. F., Roelands M. C. P., White R. J., van Harmelen T., de Wild P., van der
Laan G. P., Meirer F., Keurentjes J. T. F., Weckhuysen B. M. (2020). Beyond Mechanical Recycling: Giving
New Life to Plastic Waste. Angew. Chem., Int.
Ed..

[4] Gross R. A., Kalra B. (2002). Biodegradable Polymers for the Environment. Science.

[5] Geyer R., Jambeck J. R., Law K. L. (2017). Production, Use,
and Fate of All
Plastics Ever Made. Sci. Adv..

[6] World Economic Forum, Ellen MacArthur Foundation & McKinsey and Company. The New Plastics Economy: Rethinking the Future of Plastics, 2016; https://ellenmacarthurfoundation.org/the-new-plastics-economy-rethinking-the-future-of-plastics.

[7] MacLeod M., Arp H. P. H., Tekman M. B., Jahnke A. (2021). The Global Threat from
Plastic Pollution. Science.

[8] Häußler M., Eck M., Rothauer D., Mecking S. (2021). Closed-loop Recycling of Polyethylene-like
Materials. Nature.

[9] Eck M., Schwab S. T., Nelson T. F., Wurst K., Iberl S., Schleheck D., Link C., Battagliarin G., Mecking S. (2023). Biodegradable High-Density
Polyethylene-like Material. Angew. Chem., Int.
Ed..

[10] Ding M., Ni L., Zheng Y., Wang B., Yu C., Shan G., Bao Y., Liu J., Pan P. (2025). Hexagonal and Orthorhombic Crystal
Formations in Ethylene Glycol-Based Long-Spaced Aliphatic Polyesters
Driven by Layer Packing of Proximate Ester Groups. Macromolecules.

[11] Menges M. G., Penelle J., Fevere Le, de Ten Hove C., Jonas A. M., Schmidt-Rohr K. (2007). Characterization
of Long-Chain Aliphatic
Polyesters: Crystalline and Supramolecular Structure of PE22,4 Elucidated
by X-ray Scattering and Nuclear Magnetic Resonance. Macromolecules.

[12] Marxsen S. F., Häuβler M., Mecking S., Alamo R. G. (2020). Isothermal Step
Thickening in a Long-spaced Aliphatic Polyester. Polymer.

[13] Marxsen S. F., Häußler M., Mecking S., Alamo R. G. (2021). Unlayered–Layered
Crystal Transition in Recyclable Long-Spaced Aliphatic Polyesters. ACS Appl. Polym. Mater..

[14] Marxsen S. F., Häußler M., Eck M., Mecking S., Alamo R. G. (2023). Effect
of CH_2_ Run Length on the Crystallization Kinetics of Sustainable
Long-spaced Aliphatic Polyesters. Polymer.

[15] Zhou L., Qin P., Wu L., Li B.-G., Dubois P. (2021). Potentially Biodegradable
“Short-Long” Type Diol-Diacid Polyesters with Superior
Crystallizability, Tensile Modulus, and Water Vapor Barrier. ACS Sustain. Chem. Eng..

[16] Janani H., Marxsen S. F., Eck M., Mecking S., Tashiro K., Alamo R. G. (2024). Polymorphism and
Stretch-Induced Transformations of
Sustainable Polyethylene-Like Materials. ACS
Macro Lett..

[17] Schwab S. T., Bühler L. Y., Schleheck D., Nelson T. F., Mecking S. (2024). Correlation
of Enzymatic Depolymerization Rates with the Structure of Polyethylene-Like
Long-Chain Aliphatic Polyesters. ACS Macro Lett..

[18] Takahashi Y., Tadokoro H. (1973). Structural Studies of Polyethers,(-(CH_2_)_m_O-) n. X. Crystal Structure of Poly (ethylene oxide). Macromolecules.

[19] Schmidt-Rohr K., Hu W., Zumbulyadis N. (1998). Elucidation
of the Chain Conformation in a Glassy Polyester,
PET, by Two-Dimensional NMR. Science.

[20] Strobl, G. R. The Physics of Polymers; Springer, 1997; Vol. 2.

[21] Gedde, U. Polymer Physics; Springer Science & Business Media, 1995.

[22] Fritzsching K. J., Mao K., Schmidt-Rohr K. (2017). Avoidance of Density Anomalies as a Structural Principle
for Semicrystalline Polymers: The Importance of Chain Ends and Chain
Tilt. Macromolecules.

[23] Kaji H., Schmidt-Rohr K. (2002). Selective
Observation and Quantification of Amorphous
Trans Conformers in Doubly ^13^C-Labeled Poly­(ethylene terephthalate),
PET, by Zero-Quantum Magic-Angle-Spinning Solid-State NMR. Macromolecules.

[24] Sun Z., Yuan S., Schmidt-Rohr K. (2023). Quantification
of Large Long Periods
in Rigid Polymer Systems by ^1^H Spin Diffusion in HetCor
NMR with Heavy Peak Overlap. Appl. Magn. Reson..

[25] deAzevedo E. R., Hu W.-G., Bonagamba T. J., Schmidt-Rohr K. (2000). Principles
of Centerband-only Detection of Exchange in Solid-state Nuclear Magnetic
Resonance, and Extension to Four-time Centerband-only Detection of
Exchange. J. Chem. Phys..

[26] Maricq M. M., Waugh J. S. (1979). NMR in Rotating
Solids. J. Chem.
Phys..

[27] Hu W. G., Boeffel C., Schmidt-Rohr K. (1999). Chain Flips in Polyethylene Crystallites
and Fibers Characterized by Dipolar ^13^C NMR. Macromolecules.

[28] Janani H., Kramer C. W., Boyd N. R., Eck M., Mecking S., Alamo R. G. (2025). Crystallization Rate Minima of Aliphatic
Polyesters
Type PE-X,Y in a Wide Range of Undercooling: Role of CH_2_ Sequence Length and Layered Crystallites. Macromolecules.

[29] Schmidt-Rohr K. (2007). Simulation
of Small-angle Scattering Curves by Numerical Fourier Transformation. J. Appl. Crystallogr..

