# Supplementary material for: Interfacial and Crystalline Gauche OCH2–CH2O Layers in Biodegradable and Recyclable Polyethylene-like Polyesters Detected by Nuclear Magnetic Resonance
Source: J Am Chem Soc. 2025 Dec 2;147(50):45924–35. doi: 10.1021/jacs.5c10528 (PMC12715792; doi:10.1021/jacs.5c10528)
Supplement: Supplementary file 1 [file ja5c10528_si_001.pdf]

Supporting Information

for

Interfacial and Crystalline *Gauche* OCH_2-CH_2O Layers in Biodegradable and Recyclable Polyethylene-Like Polyesters Detected by Nuclear Magnetic Resonance

Zhenhuan Sun[1], Taylor Frederick Nelson[2], Stefan Mecking[2]*, Klaus Schmidt-Rohr[1]*

[1]: Department of Chemistry, Brandeis University, Waltham, MA 02453, USA.

[2]: Department of Chemistry, University of Konstanz, 78457 Konstanz, Germany.

Methods

Materials for polymer synthesis. Dimethyl 1,12-dodecanedioate (>98%) was purchased from TCI and used as received. Octadecanedioic acid (97%) was purchased from Elevance Renewable Sciences Inc., and used to synthesize dimethyl 1,18-octadecanedioate *via* acid-catalyzed esterification with methanol, as reported previously.[1] Dimethyl 1,48-octatetracontadioate was synthesized *via* "chain multiplication" of erucic acid (technical grade, 90%; Sigma Aldrich) as reported previously.[2] Non-labeled ethylene glycol (>99.5 %) from Carl Roth was degassed and stored under inert atmosphere prior to use. $^{13}C_2$-labeled ethylene glycol (99 atom% ^{13}C) from Sigma Aldrich was received in sealed ampules, which each contained roughly 1 g and were immediately consumed upon opening. Dibutyltin oxide (DBTO; synthesis grade) was from Sigma Aldrich. The following solvents used for the dissolution, precipitation, and washing, respectively, of polyesters: xylene (>99%, Carl Roth), isopropanol (>99%, VWR), and acetone (technical grade).

Polymer synthesis and processing. Polyesters were synthesized in a manner similar to previously reported procedures.[3] The corresponding dimethyl ester (1.0 eq.) together with ^{13}C-labeled (≥ 99 atom% ^{13}C) or unlabeled ethylene glycol (2.0 eq., usually 1 g) and DBTO as polycondensation catalyst (0.005 eq.) were added to a 25 mL Schlenk tube with an extended side arm and flask to collect the condensate. The application of excess ethylene glycol resulted in almost exclusively ^{13}C-diol end groups in the polyesters (see **Figure 1**, main text).

After repeatedly evacuating and flushing the reaction setup with inert gas, the reaction mixture was heated to 150 °C under stirring via a PTFE-coated magnetic stir-bar for 3 hours at atmospheric pressure. Then, oligomerization was continued under reduced pressure (ramping down from 800 to 10 mbar over the course of 4-6 hours) to remove condensate, followed by a polymerization phase at < 0.1 mbar overnight (14-18 hours). For PE-2*,48, only 0.5 g $^{13}C_2$-ethylene glycol was employed, and an additional final polymerization phase of 2 h at 180 °C was employed. The resulting polymer melts were immediately dissolved in hot xylene, and then precipitated in cold isopropanol, washed with acetone, and after filtering dried at 50 °C and 50 mbar. The precipitated polymers were heated to 150 °C in aluminum pans *in vacuo*, then the cooled materials were cryogenically ball milled in a Retsch Cryomill for 3 × 1 min cycles at 30 Hz, and finally the recovered powders were re-dried at 50 °C and 50 mbar.

In order to make sure that unexpected crystalline *gauche* segments in PE-2*,12 were not kinetically trapped, 60 mg of PE-2*,12 was melt-compressed manually in a 15-mm long thick-walled glass tube of 2.9 mm inner diameter. The tube with the polymer plug was placed upright in an oven, heated to 100 °C where the polymer formed a clear melt, and kept at 100 °C for 30 minutes to erase its thermal history. The temperature was then lowered to 75 °C, kept there for 20 minutes, and subsequently decreased at a controlled slow rate of 0.1 °C min^{-1} for nearly isothermal crystallization, until reaching 50 °C. Finally, the tube with the sample was taken out of the oven and cooled to ambient temperature. The solid polymer cylinder was removed from the glass tube using a small screw, wrapped in a layer of Teflon tape, and transferred into a 4-mm rotor (inner diameter of 2.9 mm).

Standard polymer characterization techniques. Room temperature size exclusion chromatography (RT-SEC) measurements were performed using a PSS SECcurity instrument (software: PSS WinGPC, version 8.32), equipped with PSS SDV linear M columns (2 × 30 cm, additional guard column) and a refractive index detector (PSS SECcurity RI). Samples were prepared in chloroform ($CHCl_3$; for HPLC, VWR) at concentrations of approx. 2 mg mL^{-1}; for analysis, 20 µL samples were injected with an applied flow rate of 1 mL min^{-1} and a column temperature of 35 °C. Molecular weights were determined versus narrow polydispersity polystyrene standards from PSS Polymer Standards.

High temperature size exclusion chromatography (HT-SEC) was performed on a Polymer Char GPC-IR instrument (software: PSS WinGPC, version 8.32), equipped with PSS Polefin Linear XL columns (3 × 30 cm, additional guard column), an infrared detector (IR5 MCT, concentration signal) and a viscosity detector. Samples were prepared in 1,2-dichlorobenzene (DCB; 99%, Thermo) at 160 °C and injected with a standard flow rate of 0.5 mL min^{-1}. Molecular weights were determined *via* universal calibration versus narrow polyethylene standards from PSS Polymer Standards.

Differential scanning calorimetry (DSC) measurements were performed using a Netzsch DSC 204 F1 instrument (software: Netzsch Proteus Thermal Analysis, version 6.1.0). For analysis, heating and cooling rates of 10 K min^{-1} were applied. Peak melting temperatures and enthalpies of melting were determined from second heating cycles recorded from -50 °C to 180 °C.

Solution NMR characterization was performed using a Bruker Avance III HD 400 spectrometer. Spectra were analyzed using Mestrenova software by Mestrelab Research S.L. (version 14.1.2). Polymers were dissolved in deuterated tetrachloroethane ($C_2D_2Cl_4$; 99.6%) from Eurisotop (stored over 4 Å molecular sieves) and spectra were collected at 373 K.

Table S1. Key physicochemical properties of synthesized polyesters.

Technique:	^{1}H NMR		HT-SEC [c]			RT-SEC [d]			DSC			ssNMR	WAXD [g]
Property:	ñ [a]	M_n [b]	M_n	M_w	Đ	M_n	M_w	PDI	T_c [e]	T_m [f]	$\Delta_m H$ [f]	χ	
Unit:		kg mol^{-1}	kg mol^{-1}			kg mol^{-1}			°C		J g^{-1}	%	
**PE-2,12**	104	13.3	4.3	12.3	2.8	18.0	37.3	2.1	63.2	85.1	87.4	60 ± 8	62 ± 4
**PE-2*,12**	115	14.8	5.3	15.6	3.0	22.3	45.5	2.0	66.3	84.8	92.6	ND	59 ± 3
**PE-2*,18**	101	17.2	9.2	23.1	2.5	32.3	67.3	2.1	79.8	97.5	122.4	57 ± 7	69 ± 3
**PE-2*,48**	58	22.3	13.8	41.5	3.0	ND	ND	ND	100.8	112.4	157.9	76 ± 5	76 ± 4

[a] Degree of polymerization, determined from end-group quantification in solution state ^{1}H NMR spectroscopy

[b] Number-averaged molecular weight, calculated from degrees of polymerization and repeat unit molecular weights of the respective polyesters

[c] Performed at 160 °C in DCB vs. polyethylene standards

[d] Performed at 35 °C in $CHCl_3$ vs. polystyrene standards

[e] Taken from 1st cooling cycle from 180 °C to -50 °C

[f] Taken from 2nd heating cycle from -50 °C to 180 °C

[g] Determined from relative contributions of peaks from crystalline domains to total integrated diffractogram areas; values represent mean ± 1 s.d. of triplicate measurements per polyester

Solid-state NMR. Modern solid-state NMR experiments were performed using a Bruker Avance Neo wide-bore 400 spectrometer, at resonance frequencies of 400 MHz for ^{1}H and 100 MHz for ^{13}C. Samples were packed into 4-mm rotors each containing a 3-mm high cylindrical glass insert and were measured in a Bruker double-resonance 4-mm probehead with high-power SPINAL-64[4] ^{1}H decoupling at $|\gamma B_1/2\pi|$ = 87 kHz. The detection period did not exceed 19 ms. The ^{13}C $\pi/2$-pulse length was 4 μs. Chemical shifts were referenced to TMS, using the COO resonance of the α-modification of glycine at 176.49 ppm as a secondary reference. In order to avoid significant spinning sidebands of the OCH_2 chemical shift anisotropy but also minimize line broadening by interference of magic-angle spinning (MAS) with radio-frequency decoupling of ^{1}H from ^{13}C, most experiments were performed at 12 kHz MAS, which places the OCH_2 spinning sideband safely beyond the ester COO peak. A spectrum of the long-T_{1C} components in PE-2,12 in natural abundance showed a smaller linewidth of the peak at 64 ppm at 6-kHz compared to 12-kHz MAS. All spectra were recorded after a rotation-synchronized Hahn spin echo (see Scheme S1), to avoid baseline distortions by pulse deadtime effects. The Hahn echo time $2\tau = 2t_r$ = 0.14 ms was so short that even a species with 3 ppm homogeneous broadening would decay only by 12%. This might apply to species with intermediate mobility, or ^{13}C spin pairs in immobile *gauche* segments. Immobile species of ^{13}C in natural abundance have a much longer T_2 values. Fully relaxed direct-polarization (DP) ^{13}C NMR spectra of PE-2*,12/18/48 were measured with recycle delays of 500 s, 200 s, and 2000 s, respectively, and are shown in Figure 1. A full series of recycle delays took around 17 hours, about half of which was used for the longest value.

The signals of CH_2 groups undergoing fast, large-amplitude motions, e.g. in the soft amorphous layers of PE-like polyesters, can be selected by dipolar dephasing, i.e. switching-off of ^{1}H decoupling for 40-70 μs, if needed with single-π-pulse recoupling.[5] Like "DipShift" methods, this experiment monitors motional averaging of the ^{13}C-^{1}H dipolar coupling, but dipolar dephasing has the advantage that the signal of immobile CH_2 groups is essentially zero and therefore does not produce an undesirable, confounding background.

In this work, the signals of immobilized *gauche* OCH_2-CH_2O segments were of particular interest. In order to separate them from the overlapping high signal of mobile *gauche* units, we generated inverse dipolar-dephased spectra by subtracting the dipolar dephased spectrum from the full spectrum. In double rigid selection, inverse dipolar dephasing was combined with short, 0.1 ms cross polarization, which prevented the CH_2 groups with motionally weakened C-H

dipolar couplings from slowly transferring magnetization from ^1H to ^{13}C. This could further be combined with a T_{1C} filter, see Scheme S1(c), where magnetization was stored alternately along +z and −z while the receiver phase is inverted accordingly,[6] of 1 – 10 s duration, which further strongly suppressed the amorphous-*gauche* signals with their short T_{1C} of 0.2 s. The spectrum of crystalline *gauche* segments, which have a long T_{1C} of 27 s, could be selected by a ≥20-s T_{1C} filter. For PE-2*,N, a full series of T_{1C} filter times as shown in Figures S1-S3 took around 3 hours to measure. A single long T_{1C}-filtered spectrum of PE-2,12 in natural abundance took 30 hours to record.

^1H spin diffusion experiments can take advantage of the pronounced mobility and T_{2H} difference between the soft amorphous layers on the one hand and the crystalline and interfacial layers on the other, to generate a steep magnetization gradient. A T_{2H} filter, see Scheme S1d, of 130 μs duration on resonance at 1.5 ppm was used to selectively retain the ^1H magnetization of the mobile CH_2 groups in the amorphous layers of PE-2*,12 and PE-2*,18, while the limited amorphous-layer mobility in PE-2*,48 required a shorter filter time of 15 μs. The magnetization was stored along the z-direction for ^1H spin diffusion to gradually erase the magnetization gradients, for durations up to T_{1H}. Subsequently, cross polarization was used to transfer the magnetization to ^{13}C, where it was detected after an optional T_{1C} filter (of 10 s duration for PE-2*,12) and a Hahn spin echo, possibly with dipolar dephasing, see Scheme S1d. The number of ester layers in the simulations of spin diffusion in PE-2*,12 and PE-2*,18 was confirmed by simulations[7] of the mid-angle X-ray scattering peaks near 0.38 and 0.28 $Å^{-1}$, respectively, as described below. A series of spin diffusion experiments typically required 22 hours.

Centerband-only detection of exchange (CODEX) NMR, see Scheme S1e, was performed at 6 kHz MAS with a total of $N = 6$ rotation periods of chemical-shift-anisotropy (CSA) recoupling, after 0.1 ms CP. The relatively low spinning frequency is favorable because it reduces the number of π-pulses for a given recoupled period Nt_r and speeds up ^{13}C-^{13}C spin exchange. The intensity S of the stimulated echo of the recoupled CSA was recorded as a function of the exchange mixing time over 4.5 orders of magnitude, from 0.2 ms to 9 s, relative to the full signal S_0 after a minimal mixing time of 0.2 ms. The fastest exchange process, on the 3-ms time scale, is one-bond ^{13}C-^{13}C spin exchange. In $^{13}CH_2$-$^{13}CH_2$ *gauche* segments, the two instantaneous anisotropic chemical shifts are generally very different, so dipolar spin exchange results in a fast drop in CODEX intensity, to an asymptotic value of ½ for this two-site

exchange,[8] while in *anti* segments, the two ^{13}C chemical shifts are similar and therefore little exchange occurs. A typical series of CODEX experiments took 24 hours; PE-2,48 required longer measurement times due to pronounced signal loss by ^{13}C spin-lattice relaxation at the longest mixing times. Two time points from unlabeled PE-2,12 took 14 hours to record.

A reviewer expressed concerns as to how spin exchange could occur in the presence of chemical-shift differences. Given that the isotropic chemical-shift differences are smaller than the ^{13}C-^{13}C and ^{13}C-^{1}H dipolar couplings, our experimental conditions can be considered as MAS at the "n = 0 rotational resonance" assisting ^{13}C spin exchange, at a moderate spinning frequency so that MAS does not average the non-commuting dipolar couplings and chemical-shift difference to zero. Magic-angle spinning results in identical chemical shifts (MAS-induced level crossings) of the coupled spins twice or more during a rotation period, so around those times, there is no truncation of the dipolar coupling and particularly effective spin exchange. It should also be noted that the one-bond spin exchange on the 3-ms scale is nearly ten times slower than the inverse of the dipolar coupling, so it is indeed not as fast as in the strong-coupling limit, but this is not a limitation in any way because CODEX can access 1000-fold longer times. Comparison with CODEX exchange rates in various molecular crystals and a polymer with much larger chemical shift differences and smaller coupling[9] shows that our present CODEX data are not at all surprising.

A T_{1C} filter right after cross polarization and before the CODEX pulses, see Scheme S1e, was used to select components with long T_{1C} without blurring of assignments by ^{13}C spin exchange. It had a duration of 10 s for PE-2*,12 and 5 s for PE-2,12 in natural abundance.

CODEX ^{13}C NMR data of the O-^{13}C-^{13}C-O segments were simulated as products of three exponential decays to non-zero final values as follows:

$$\frac{S(t)}{S_0(t)} = \left(a + (1-a)\exp\left(-\frac{t}{T_a}\right)\right) \cdot \left(b + (1-b)\exp\left(-\frac{t}{T_b}\right)\right) \cdot \left(c + (1-c)\exp\left(-\frac{t}{T_c}\right)\right)$$

The time constants $T_a < T_b < T_c$ were different by more than a factor of five. For immobilized *gauche* conformers in PE-2*,12 and PE-2*,48, as expected $a = 0.500 \pm 0.025$, and $a = 0.59$ for interfacial *gauche* segments in PE-2*,18 (probably due to some mobility), while $a \geq 0.95$ for *anti* conformers. The constant b ranged between 0.5 and 0.68. The third constant c was allowed to freely vary between 0 and 1.

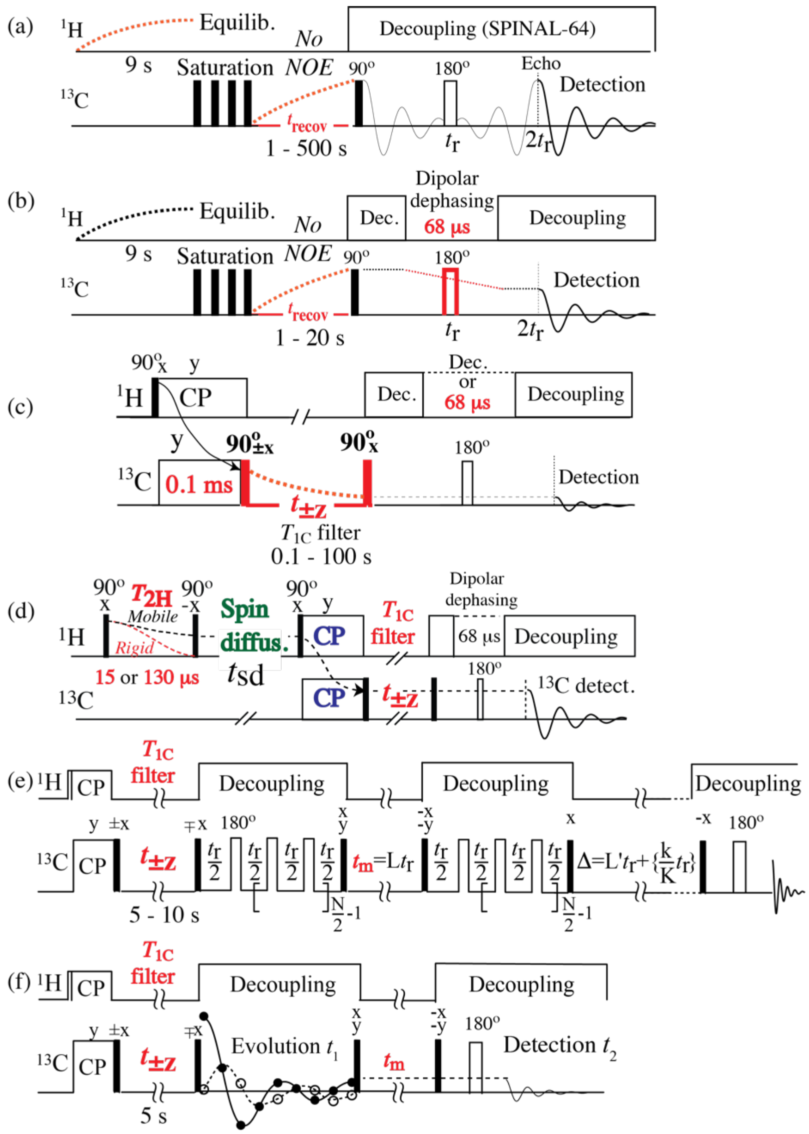

Scheme S1. Solid-state NMR pulse sequences used in this work. 90° pulses are shown as filled black rectangles. The rotation period is denoted as t_r.

(a) Direct-polarization (saturation-recovery) ^{13}C NMR without heteronuclear nuclear Overhauser enhancement (NOE) distorting peak intensities and with a rotation-synchronized Hahn echo with EXORCYCLEd[10] π-pulse before detection (in Figs. 1, S4, S6, and S7).

(b) Direct-polarization ^{13}C NMR without heteronuclear NOE and with 68-µs recoupled dipolar dephasing[5] (in Figs. 1, S4, S6, and S7). Inverse dipolar dephasing refers to the difference of the spectra obtained with sequences a) and b).

(c) Short (0.1 ms) cross polarization plus T_{1C} filter without (dashed) or with 68-µs recoupled dipolar dephasing (in Figs. 2, 3, 4, 5, and S1). The difference of the spectra obtained with the two variants is inverse dipolar dephasing (selection of immobile components).

(d) T_{2H} filter followed by ^{1}H spin diffusion, plus T_{1C} filter with dipolar dephasing (in Fig. 6).

(e) T_{1C}-filtered CODEX[8] (in Fig. 7) for determining the OCH_2-CH_2O torsion angle of immobilized segments, without scrambling of magnetization by spin exchange during the T_{1C} filter.

(f) T_{1C}-filtered 2D exchange ^{13}C NMR[11] (in Fig S9).

Numerical simulations of ^{1}H spin diffusion with diffusion coefficients ranging from 0.1 to 0.8 nm^2/ms (estimated from component mobility and ^{1}H density) were performed on 1D grids with grid spacings of ~0.04 nm using MATLAB. Reflecting boundary conditions were implemented at the ends of the grids. The time step was 0.5 μs, and a total of 800 ms were simulated. The layout and the ratio of each simulated component (amorphous, interfacial & crystalline OCH_2, and crystalline CCH_2) for each sample were constrained by X-ray diffraction measured crystal sizes as well as ssNMR measured compositions. The magnetization-sink curves were normalized by dividing by the last simulated data point. The magnetization-source curve as shown in the PE-2,48 simulation (Figure 11) was normalized by dividing by the first data point. The simulation of PE-2*,18 includes a thin outer interfacial layer (shown in yellow in Figure 9) thought to consist of less mobile α-CH_2 and COO groups, which is therefore neither part of the source of the magnetization (selected by a hard T_{2H} filter) nor of the detected interfacial OCH_2 segments.

Quantum chemical calculations were conducted using Gaussian 16.[12] To perform dihedral angle scans, corresponding dihedral angles (OCCO, CCOC, OCCC) of each molecular chain were frozen and then geometrically optimized with M062X/6-31+G(d,p). The energy as well as the ^{13}C magnetic shielding principal values of the optimized molecular chains were calculated with mPW1PW91/6-311+G(2d,p) using the gauge including atomic orbitals (GIAO) method. The magnetic shielding values were converted to ^{13}C chemical shifts using scaling factors published by Lodewyk et al.[13] Specifically, for Opt=M062X/6-31+G(d,p) and NMR=mPW1PW91/6-311+G(2d,p) in the gas phase, the formula used to convert ^{13}C calculated isotropic magnetic shieldings σ_{iso} to isotropic chemical shifts δ_{iso} is $\delta_{iso} = \frac{185.6582-\sigma_{iso}}{-1.0221}$.

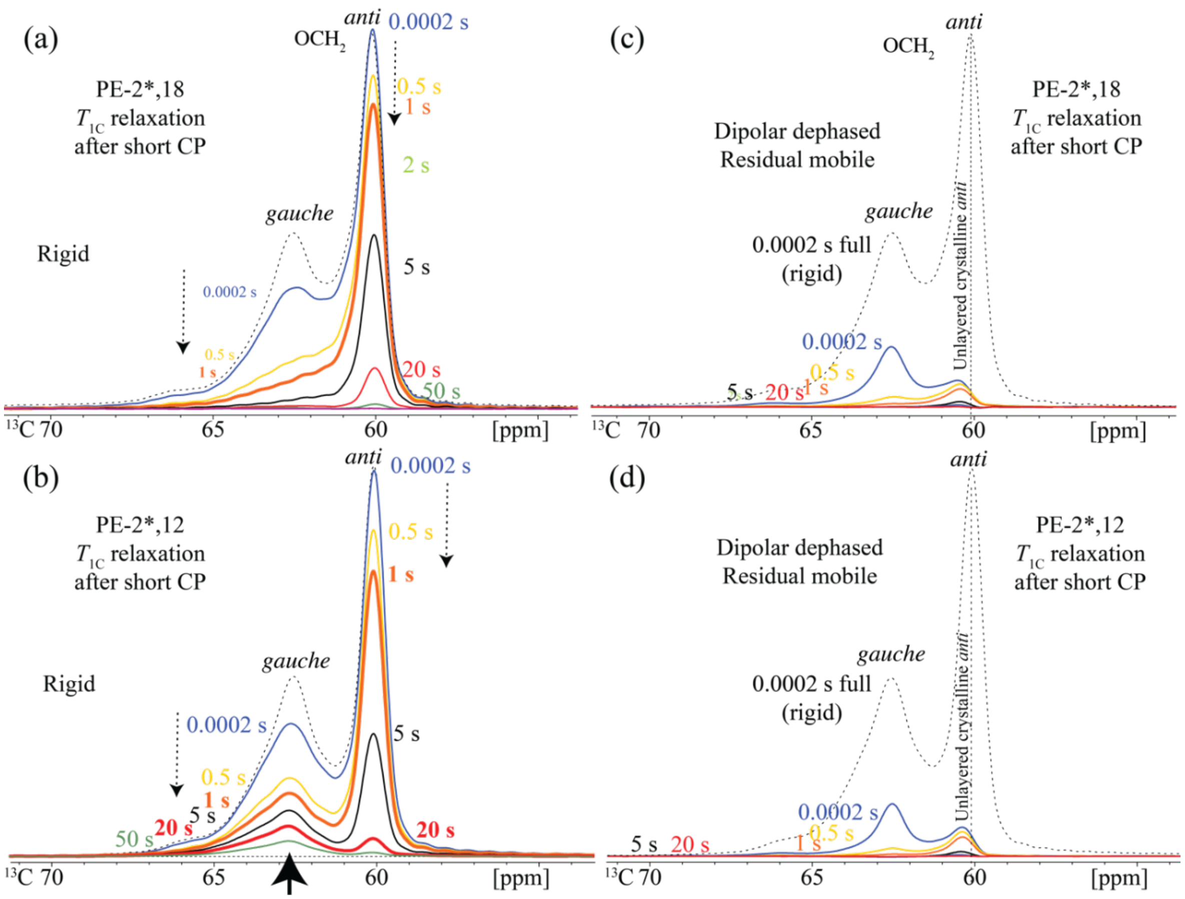

Figure S1. T_{1C} relaxation of rigid components (after short 0.1-ms CP) in (a, c) PE-2*,18 and (b, d) PE-2*,12 after (c, d) regular and (a, b) inverse dipolar dephasing. The black arrow marks the rigid *gauche* components with unusually slow T_{1C} relaxation. Compare Figure 2.

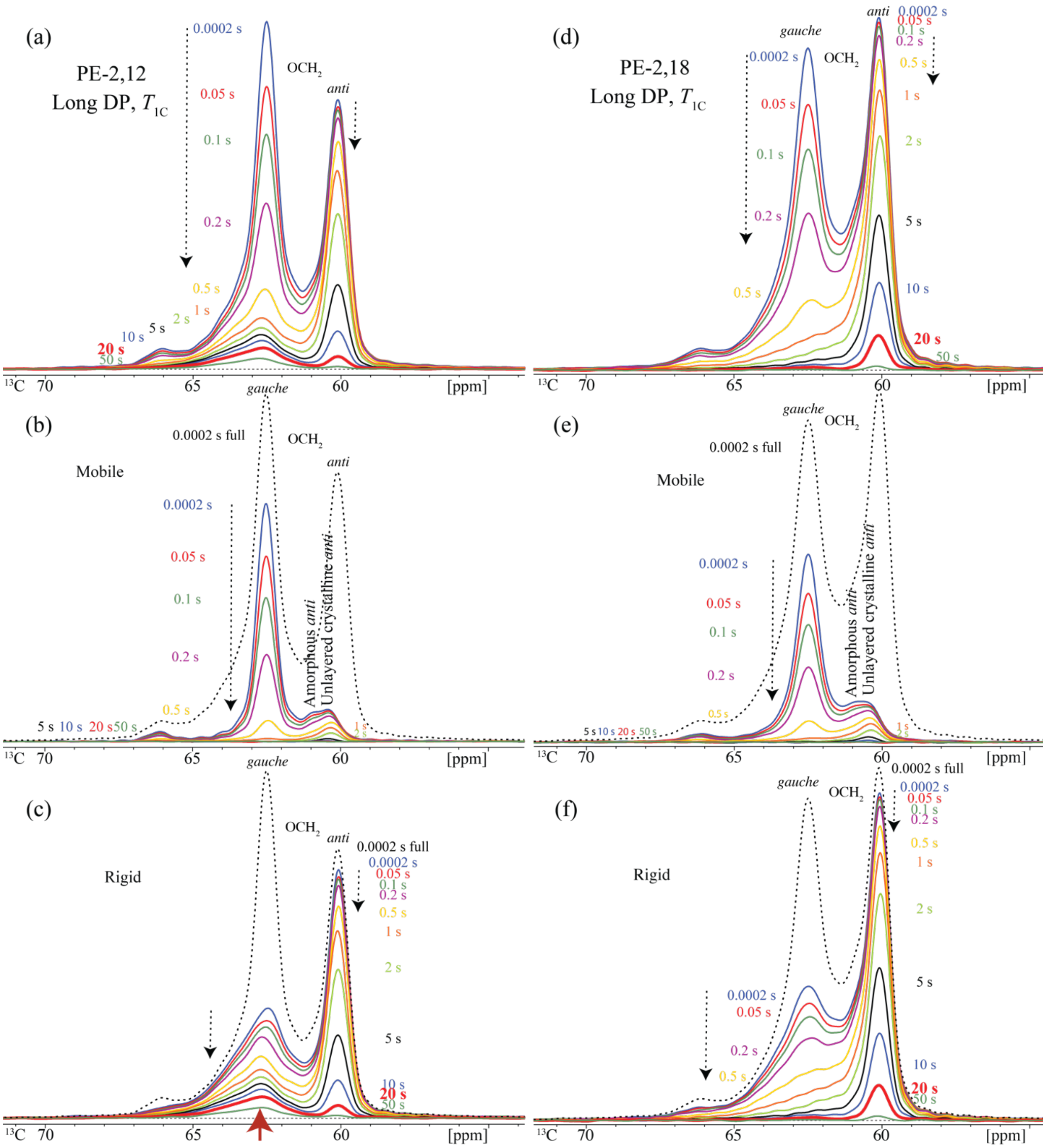

Figure S2. Spectrally resolved T_{1C} relaxation in (a-c) PE-2*,12 and (d-f) PE-2*,18, after full relaxation and direct polarization followed by a ±z-filtered T_{1C} relaxation period of the indicated duration. (a, d) Spectra of all carbons and (b, e) of mobile segments after dipolar dephasing. (c) Difference between a) and b), termed “inverse dipolar dephasing”; the red upward arrow marks the slowly relaxing *gauche* component. (f) Difference between d) and e), with nearly insignificant slowly relaxing *gauche* component. Compare Figure 2.

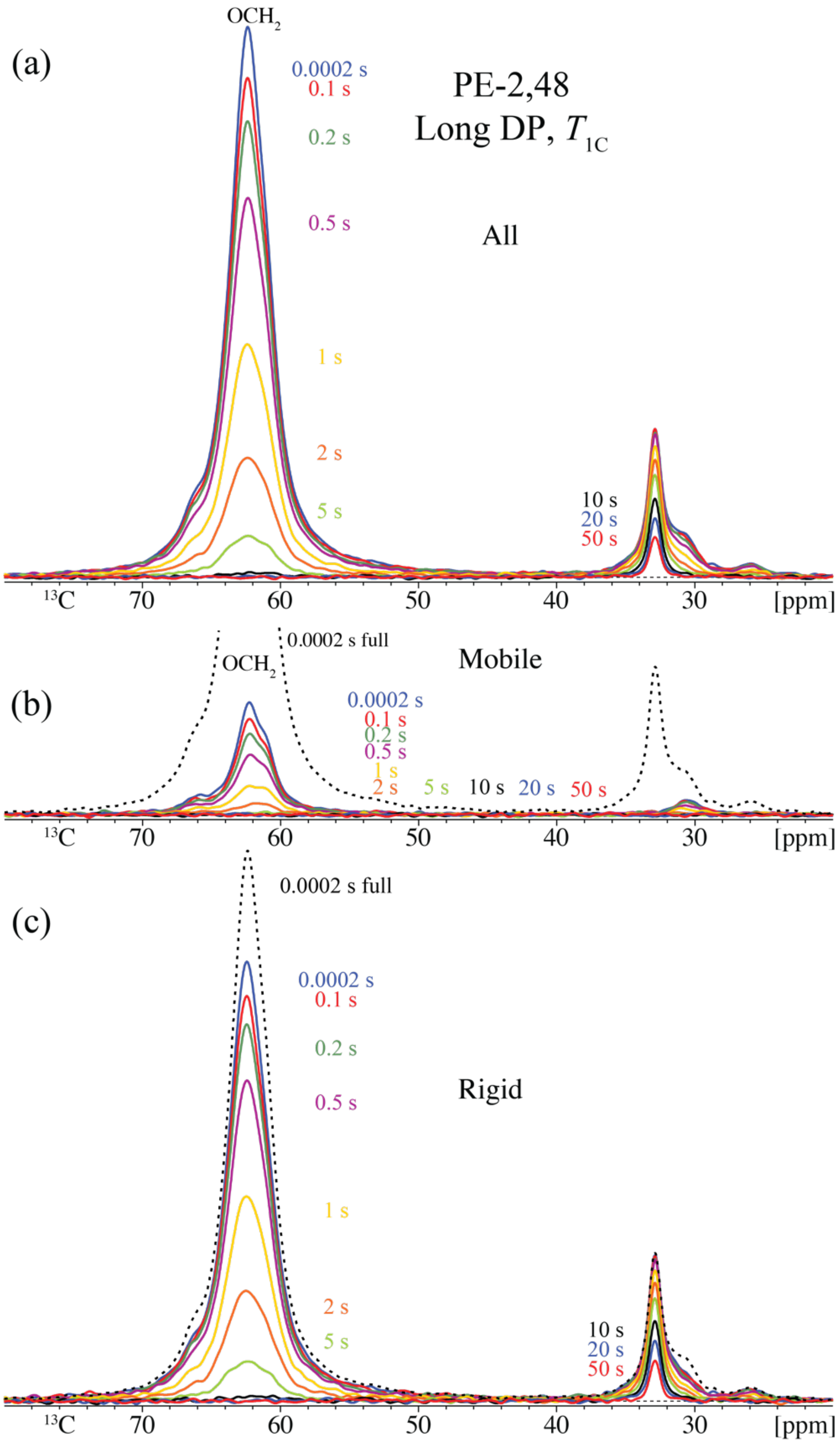

Figure S3. Spectrally resolved T_{1C} relaxation in PE-2*,48, after full relaxation and direct polarization followed by a ±z-filtered T_{1C} relaxation period of the indicated duration. (a) All carbons; (b) mobile segments after dipolar dephasing, a minor fraction. (c) Difference between a) and b): "inverse dipolar dephasing". Compare Figure 5.

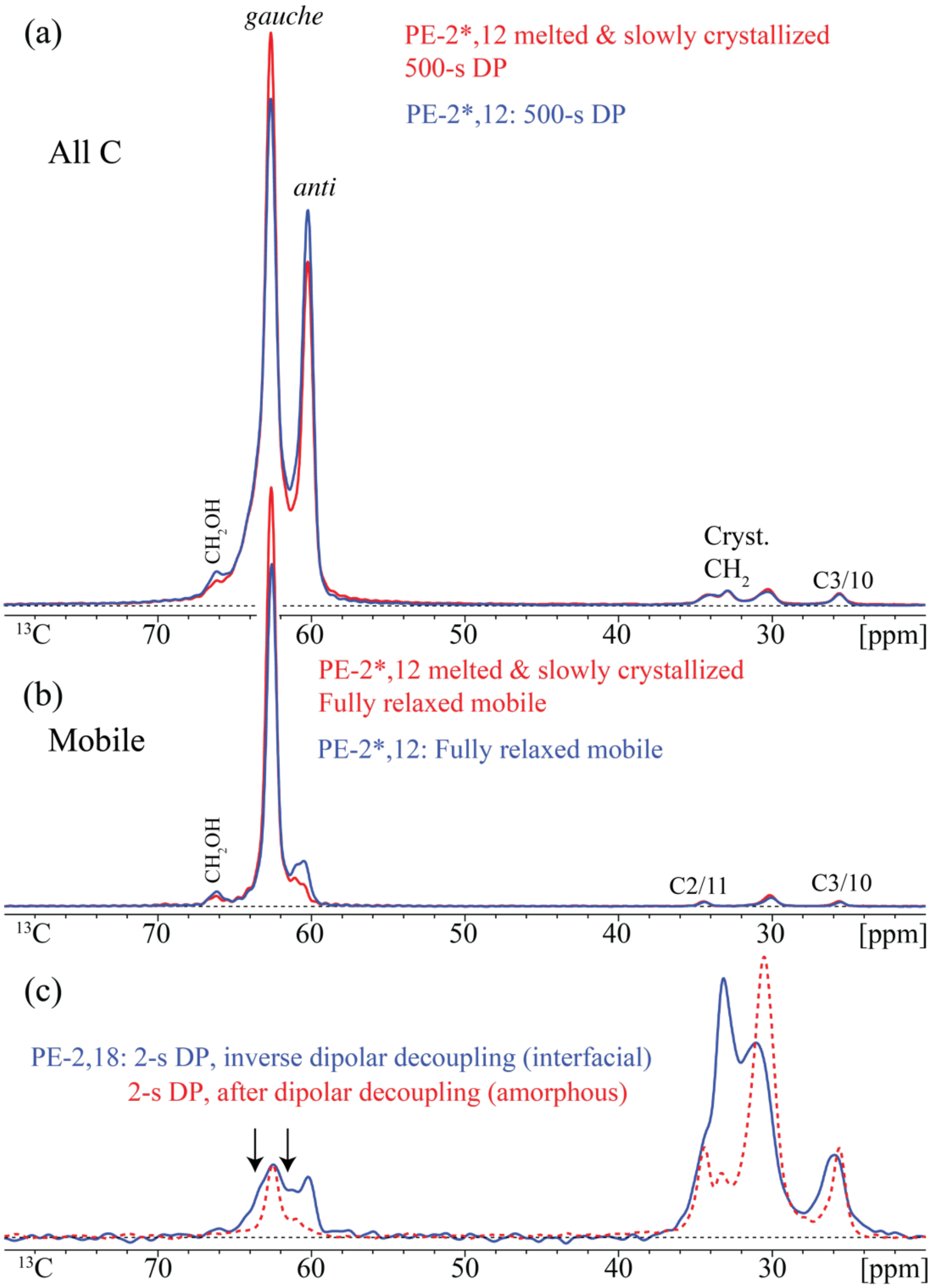

Figure S4. (a, b) Comparison of the quantitative ^{13}C NMR spectra of PE-2*,12 (blue traces) and melted and slowly recrystallized PE-2*,12 (red traces). (a) All carbons; (b) mobile segments after recoupled dipolar dephasing. (c) Direct-polarization spectra of PE-2,18 (unlabeled) with 2-s recycle delay without NOE (see Scheme 1a), after regular (red dashed) and inverse (blue) dipolar dephasing, documenting (inhomogeneous) broadening of the OCH_2 signal of the immobilized interfacial components (marked by arrows) even in the absence of ^{13}C-labeling.

MAXS peak simulations. The morphologies of PE-2,18 and PE-2,12, specifically the number of diol layers in a typical crystalline lamella, can be assessed by comparing the reported[14,15] mid-angle X-ray scattering (MAXS) peaks near 0.28 and 0.38 $Å^{-1}$, respectively, with simulations for various layered models. The larger the number of layers, the narrower the MAXS peak. Simulations assuming indefinitely wide, parallel crystalline lamellae were performed using the algorithm of ref.[7] with elementary box form factors. The simulated electron density perpendicular to the normal to the lamellae consists of ~10^5 crystalline lamellae alternating with amorphous layers of a relative electron density of 0.95 and an average thickness that produced a crystallinity of 65%. The amorphous-layer thickness had a Gaussian distribution with σ as one quarter of the average thickness and truncated at ±2.5 σ to maintain a minimum thickness. Without this random variation, the MAXS peak shows a fine modulation with a period of $2\pi/L_P$, where L_P is the long period of the system. A crystallite usually consisted of n diester-and-diol layers, each with a thickness of 9 grid points (0.45 nm) and a relative electron density of 1.15, alternating with n-1 layers of crystalline $C(CH_2)_{N-2}C$ segments with a length of 2N grid points (0.1 N nm, e.g. 1.8 nm for PE-2,18) and a relative electron density of 1.

PE-2,18 models with n = 2 to 5 and 10 were simulated and produced MAXS peaks centered at $q_{max} = 2\pi/(22.5\ Å) = 0.28\ Å^{-1}$ with decreasing finite-size broadening as the number of layers in each crystal increased, see red traces in Figure S5a-e. For comparison, the scattered intensity from the same morphology but without modulation by diester/diol layers and the densities of the alternating ester and crystalline ethylene unit region averaged out instead, is also shown (blue traces in Figure S5). At integer multiples of q_{max}, there are smaller peaks (not shown), as expected since the density modulation is not sinusoidal. The system with n = 2 corresponds to a model where the amorphous ethylene units are followed immediately by half-length crystalline ethylene units instead of ester units, and the entire repeating unit is 15% thicker than usual since no chain tilt was assumed. A mixed model of PE-2,18 with crystallites 4 or 3 layers thick was also simulated, see Figure S5f.

Finally, Figure S5g shows simulated X-ray scattering patterns of PE-2,12 with four $C(CH_2)_{10}C$ layers in a crystallite. Compared to the simulations of PE-2,18, the MAXS peak[15] is moved to higher q values because the spatial period is shorter in PE-2,12 than in PE-2,18.

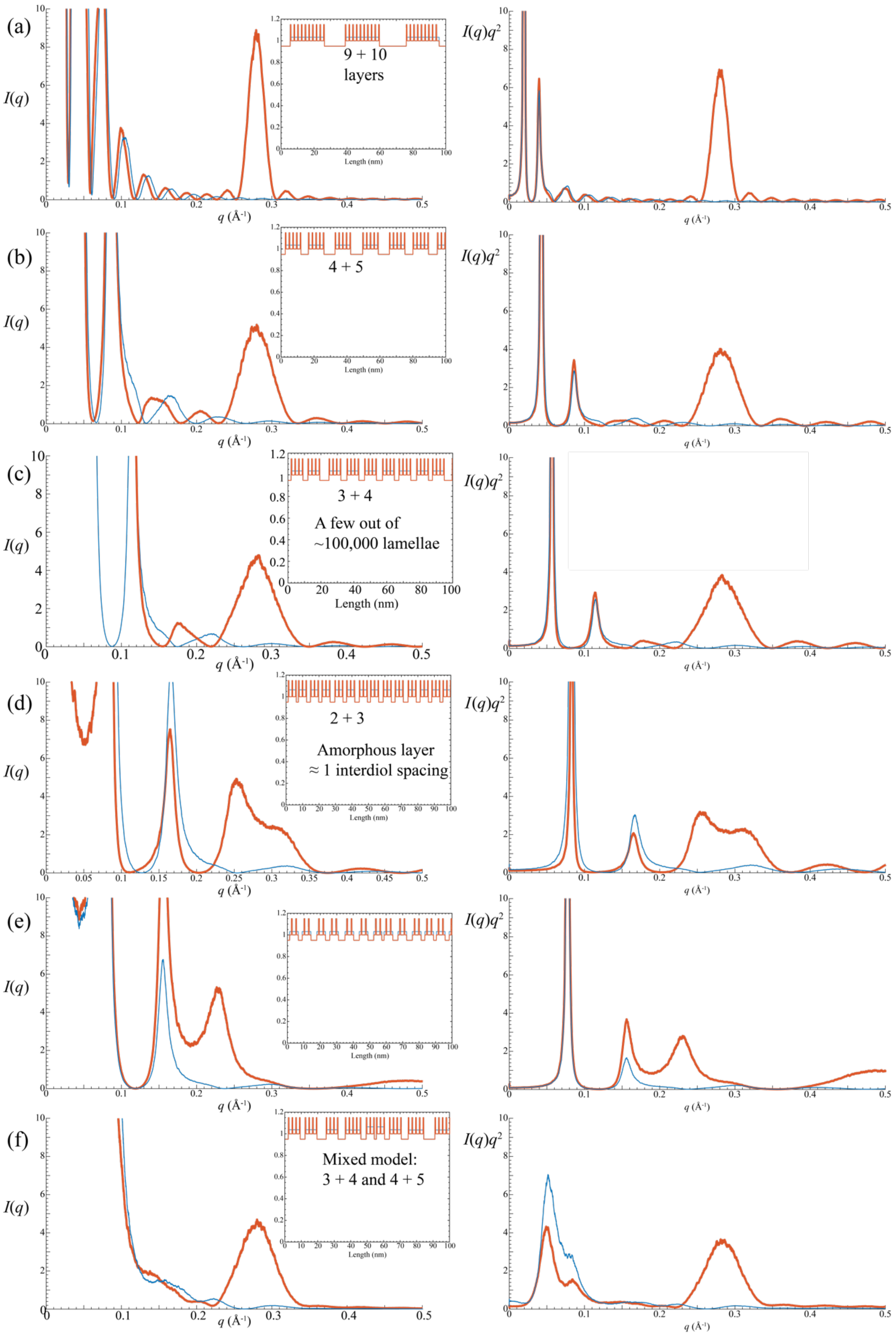
(a)
I(q)
q (Å⁻¹)
9 + 10
layers
Length (nm)
I(q)q²
(b)
4 + 5
(c)
3 + 4
A few out of
~100,000 lamellae
(d)
2 + 3
Amorphous layer
≈ 1 interdiol spacing
(e)
(f)
Mixed model:
3 + 4 and 4 + 5

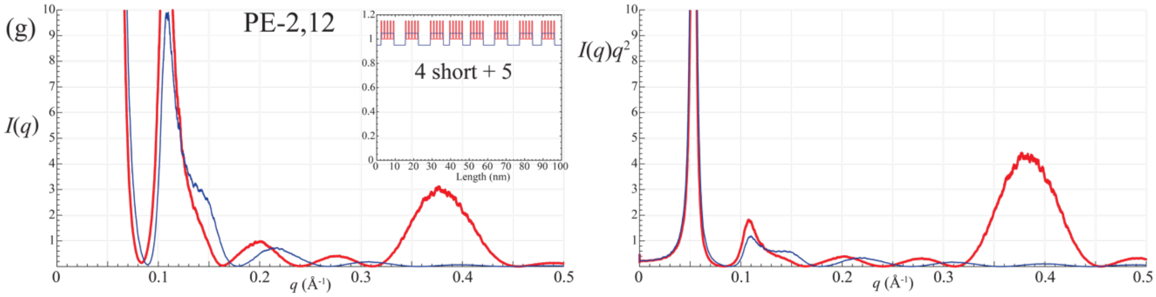

Figure S5 (previous page and above): (a-f) Simulated X-ray scattering patterns of PE-2,18 (left column: $I(q)$; right column: $I(q)\ q^2$) for the layered polyester structures whose scattering density profiles are shown in the insets. The number of $C(CH_2)_{16}C$ layers in a crystallite was n−1 = (a) 9; (b) 4; (c) 3; (d) 2; (e) 1; the number n of diol plus diester layers is larger by one. "9 + 10 layers" means that a crystalline lamella contains 9 $C(CH_2)_{16}C$ layers and 10 diol/diester layers (including the interfacial layers). (f) shows a mix of 3 and 4 $C(CH_2)_{16}C$ layers in a crystallite. The width of the MAXS peak near 0.28 $Å^{-1}$ in c) and f) best matches experimental results reported in the literature[14]. (g) Simulated X-ray scattering patterns of PE-2,12 with four $C(CH_2)_{10}C$ layers in a crystallite, with the MAXS peak[15] moved to higher q values because the spatial period is shorter in PE-2,12 than in PE-2,18.

$(CH_2)_n$ NMR signals. Between 25 and 40 ppm, the natural-abundance signals of CH_2 groups not bonded to oxygen can be observed, see Figures S6 and S7. While for PE-2,48, an all-*anti* $(CH_2)_n$ peak near 33 ppm and a broader band of mobile segments near 31 ppm are observed similarly as in high-density PE, the intensity pattern for PE-2*,12 in Figure 1a does not resemble that of PE, seeming to show relatively little *anti* signal near 33 ppm. However, the limited signal intensity is expected, as only the four middle carbons C5-C8 (δ and ε) have PE-like chemical shifts unperturbed by proximity of the ester groups. For all other carbons (C2/11 or α, C3/10 or β, and C4/9 or γ), signals from the crystalline and amorphous regions more or less coincide and therefore produce relatively high peaks. For C5-C8, *anti* peaks near 33 ppm and *gauche* signals near 30.5 ppm are separated, and consequently the signal at 33 ppm does not stand out much. At a crystallinity of around 57%, the 33-ppm peak of the crystalline δ and ε carbons is comparable in height to the crystalline plus amorphous α peak to its left.

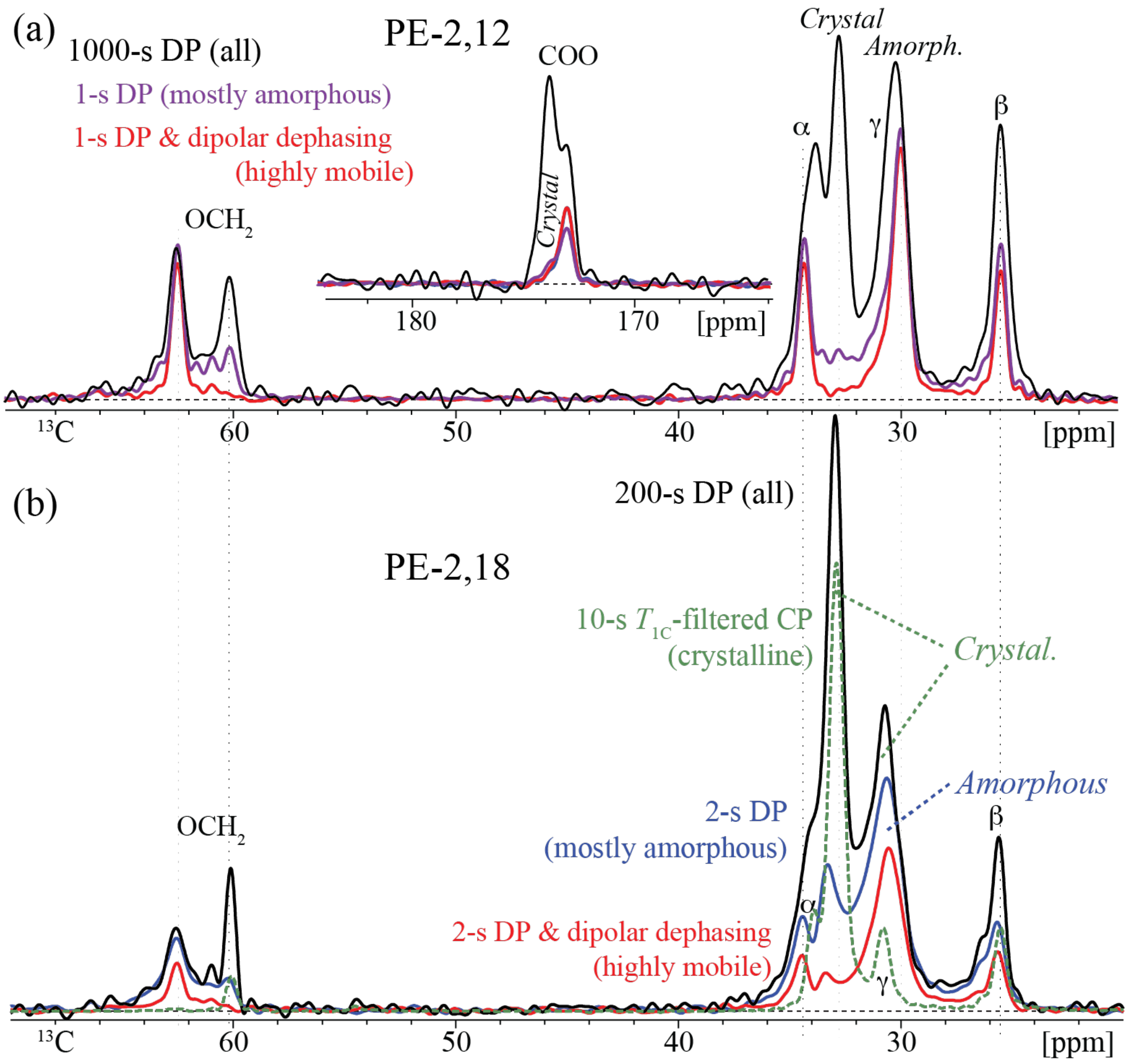

Figure S6. ^{13}C NMR spectra for crystallinity determination in (a) PE-2,12 and (b) PE-2,18 (without ^{13}C labeling). Black line: Full, quantitative ^{13}C NMR spectrum after full relaxation and direct polarization (DP); blue: DP with 2-s saturation-recovery delay, suppressing slowly-relaxing crystalline signals; red: spectrum of mobile segments after 1- or 2-s DP and dipolar dephasing; purple; DP with 1-s saturation-recovery delay. In order to avoid intensity distortions by heteronuclear Overhauser enhancement, all DP spectra were measured with ~9 s of full pre-relaxation of protons before ^{13}C saturation and the quoted saturation-recovery delay (see Scheme S1a). Dashed green: spectrum of crystalline components after a 10-s T_{1C} filter for reference. Total experiment time per sample: 28 hours.

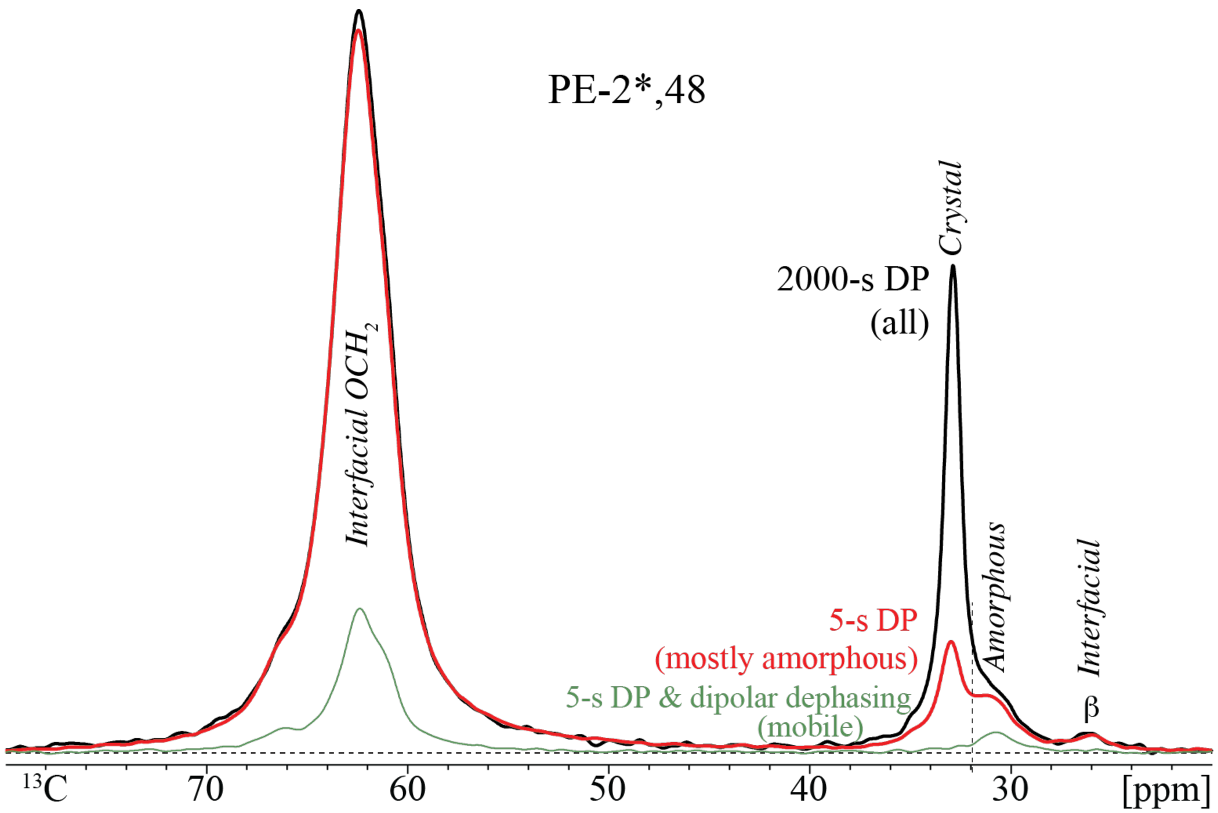

Figure S7. ^{13}C NMR spectra for crystallinity determination in PE-2*,48. Black line: Full, quantitative ^{13}C NMR spectrum after full relaxation and direct polarization (DP); red: DP with 5-s saturation-recovery delay, suppressing slowly-relaxing crystalline signals; thin green line: spectrum of mobile segments after 5-s DP and dipolar dephasing; purple; DP with 5-s saturation-recovery delay. In order to avoid intensity distortions by heteronuclear Overhauser enhancement (NOE), the spectra were measured with 9 s of full pre-relaxation of protons to equilibrium, which prevents NOE of ^{13}C, before ^{13}C saturation and the quoted saturation-recovery delay.

Crystallinity determination by NMR. Crystallinity is an important characteristic of the PE-like polyesters studied here. Since their morphology contains three types of layers: crystalline, amorphous and interfacial, the reported crystallinity may differ whether it is inclusive or exclusive of the interfacial layer; including half of each interfacial layer in the crystallinity and half in the amorphous fraction seems advisable.

Since the interfacial layers formed by the diol esters contain few $(CH_2)_n$ units, crystallinity derived from the $(CH_2)_n$ peak intensities is not affected by the interfacial layers. The amorphous fraction α, which gives the crystallinity according to $\chi = 100\% - \alpha$, can then be

estimated using the method of two DP spectra[16] as shown in Figures S6 and S7: After a few seconds of saturation recovery, the spectrum is dominated by the signals of the mobile amorphous segments so that the amorphous fraction can be quantified; the intruding small signal from PE-like crystallites near 33 ppm is adequately resolved to be excluded from the amorphous-signal integral. The full signal after relaxation of all components to equilibrium, achieved within 200 – 2000 s depending on the material, provides the 100% reference. In order to avoid distortion by heteronuclear NOE, the ^{1}H magnetization was pre-relaxed to equilibrium for 9 s, see Scheme S1a. The selection of mobile segments by a short recycle delay can be combined with recoupled dipolar dephasing, see Scheme S1b, in order to assess the detected segments' motional amplitude. For PE-2,12, the spectra in Figure S6a reveal that broad and low signal between the sharp peaks of highly mobile methylene units is from segments of limited mobility, some of which are interfacial if not crystalline. The pronounced signal loss observed for PE-2,48, see Figure S7, is characteristic of limited mobility and distinctly different from the minimal dephasing of the sharp peaks in PE-2,12. This indicates thinner, more constrained amorphous layers in PE-2,48 than in PE-2,12, consistent with other observations.

Crystallinity information can also be gleaned from the COO line shape in PE-2,12, which shows two partially merged peaks, see the inset in Figure S6a. The right peak relaxes much faster and can therefore be attributed to the mobile amorphous regions. The crystalline:amorphous peak ratio is about 60:40.

Alternatively, the amorphous fraction and corresponding crystallinity can be determined from the equilibrium amorphous magnetization percentage in ^{1}H spin diffusion out of the amorphous layers. The data in Figure 6 show decays of the sharp amorphous OCH_2 peaks of PE-2*,12 and -2*,18 to below 50%. In Figure 11, for PE-2,48, the final value of the amorphous OCH_2 fraction (selected by dipolar dephasing) is 25%, which is in excellent agreement with the completely independent spectroscopic value of 24% obtained from the data in Figure S7.

The crystallinities of PE-2,12 and PE-2,18 from NMR, 60±8% and 57±7%, respectively, see Table S1, are in fairly good agreement with the SAXS crystallinities of 57% and 55%, respectively, obtained as the ratio of crystallite thickness to long period.[14] Determination of crystallinity (and crystallite thickness) by DSC is difficult for these polyesters because the *gauche* diol surface layers documented here greatly alter the surface energy, and the esters in the crystallites affect the cohesive energy.

Crystallinity determination by WAXD. Crystallinities determined from NMR are in good to fair agreement with those determined empirically from WAXD (see Table S1). Here, each polyester's crystallinity extent was calculated from the relative contribution of the integrated areas of crystalline peaks to the total integrated area of t he diffractogram, as follows:

$$\chi_{WAXD}(\%) = \left(A_{c[110]} + A_{c[200]}\right) / \left(A_{c[110]} + A_{c[200]} + A_a\right)$$

where $A_{c[110]}$ and $A_{c[200]}$ are the areas of peaks corresponding to the Bragg reflections from the orthorhombic polyethylene-like crystallites, and A_a is the area of the peak corresponding to the amorphous halo. Peak areas were determined by deconvolution of the diffractogram using a Voigt fit via Origin 2025 (version 10.2.0.188). Figure S8 shows measured diffractograms, including fitted peak deconvolution, for each polyester.

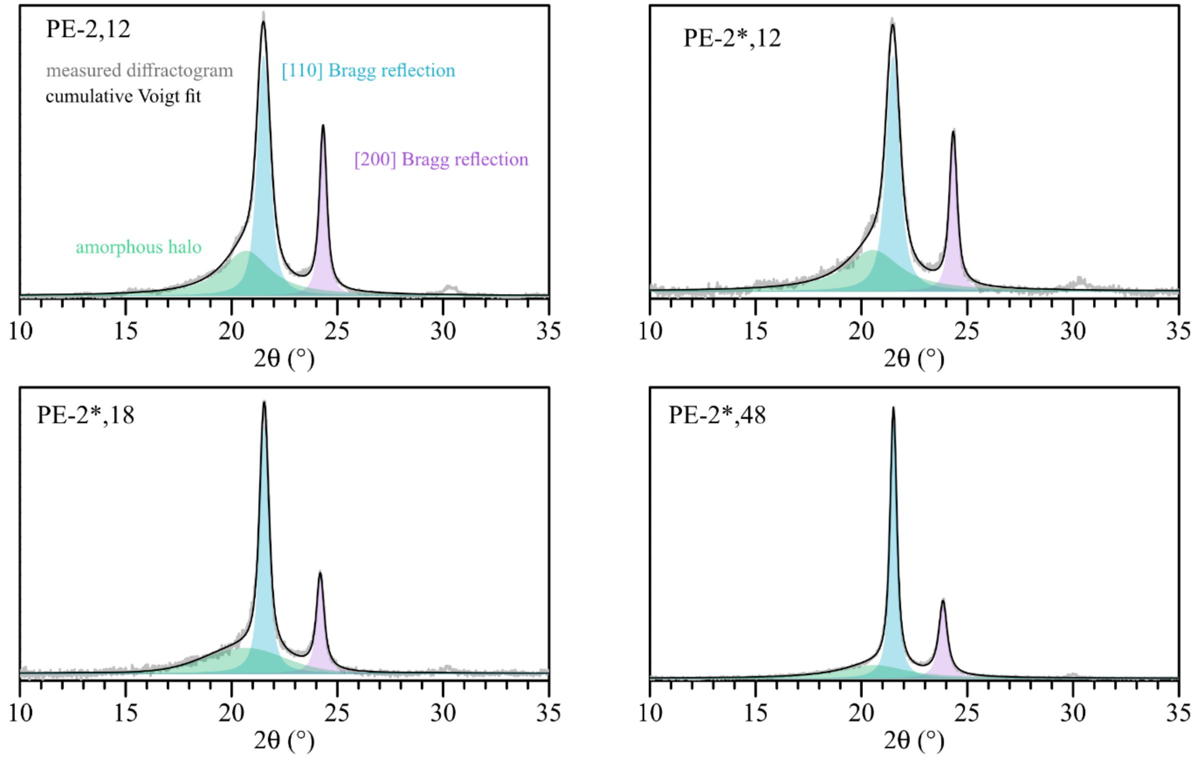

Figure S8. WAXD diffractograms of the polyesters studied here. Gray lines: measured intensity; black lines: cumulative Voigt fit of diffractogram; green, blue, purple areas: areas of deconvoluted peaks corresponding to the [110] Bragg reflection, [200] Bragg reflection, and amorphous halo, respectively.

OH chain ends. Many of the ^{13}C NMR spectra shown exhibit a foot or small peak near 66 ppm. It can tentatively be assigned to CH_2OH chain ends. Its intensity corresponds to ~3% of two of 24 heavy atoms in PE-2,18, each associated with 14 g/mol. The corresponding number average molecular weight is M_N ~ 10 kg/mol, agreeing reasonably well with the M_N ~ 17 kg/mol determined from end group analyses using solution-state ^{1}H NMR (see Table S1). This signal near 66 ppm has multiple components. A sharp peak is associated with reduced dipolar couplings and increased mobility, but in Figure 2, a less distinct, broader component is seen to be immobilized with moderate T_{1C}, suggesting an interfacial location [17].

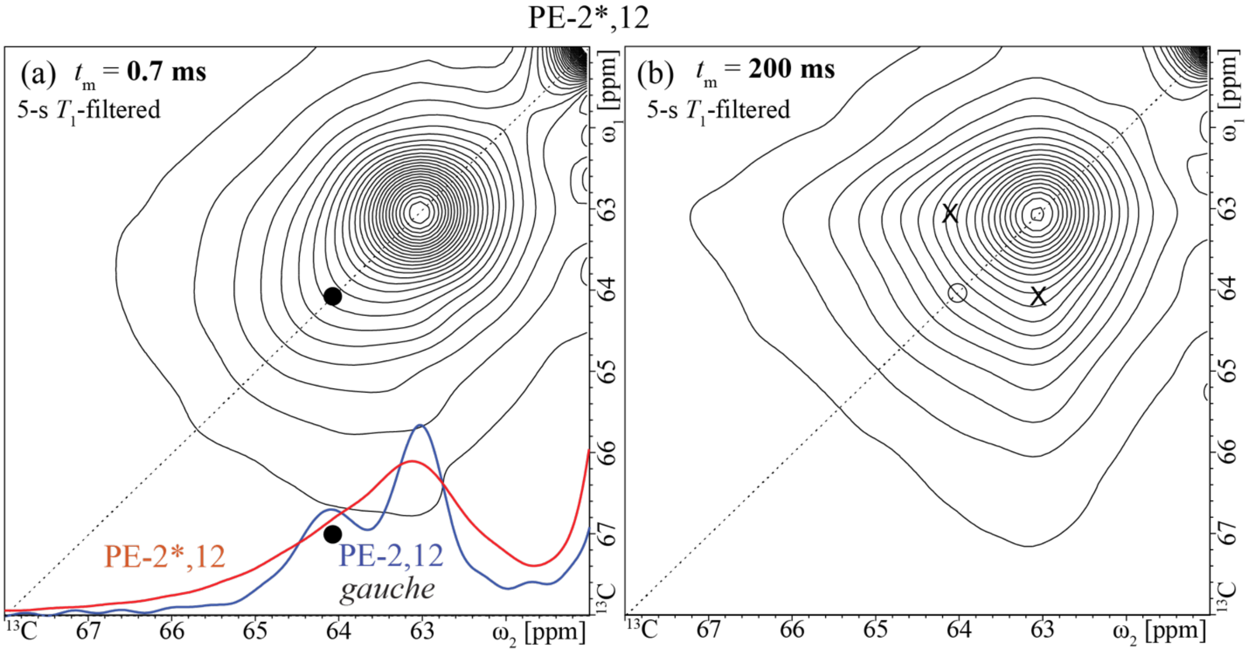

Figure S9. 2D exchange ^{13}C NMR of the crystalline *gauche* peaks of PE-2*,12, with (a) a very short mixing time of 0.7 ms, for reference; (b) a mixing time of 200 ms, sufficiently long to allow for dipolar spin exchange between directly bonded OCH_2 segments. In (a), the two crystalline *gauche* peaks observed in the 1D spectrum of unlabeled PE-2,12 (blue trace) are compared with the spectrum of PE-2*,12 (red trace) broadened by homonuclear couplings. In (b), suspected cross peaks are marked by "X". Noncrystalline signal was suppressed by a 5-s T_{1C} filter directly after short cross polarization of 0.1 ms. Spinning frequency: 14 kHz; combined measurement time: 23 hours.

References

1. Häußler, M., Eck, M., Rothauer, D., and Mecking, S., *Closed-loop Recycling of Polyethylene-like Materials.* Nature **2021**. 590(7846) 423-427.DOI: 10.1038/s41586-020-03149-9.
2. Witt, T., Häußler, M., Kulpa, S., and Mecking, S., *Chain Multiplication of Fatty Acids to Precise Telechelic Polyethylene.* Angew. Chem. Int. Ed. **2017**. 56(26) 7589-7594.DOI: 10.1002/anie.201702796.
3. Nelson, T.F., Rothauer, D., Sander, M., and Mecking, S., *Degradable and Recyclable Polyesters from Multiple Chain Length Bio- and Waste-Sourceable Monomers.* Angew. Chem. Int. Ed. **2023**. 62(43) e202310729.DOI: 10.1002/anie.202310729.
4. Fung, B.M., Khitrin, A.K., and Ermolaev, K., *An Improved Broadband Decoupling Sequence for Liquid Crystals and Solids.* J. Magn. Reson. **2000**. 142(1) 97-101.DOI: https://doi.org/10.1006/jmre.1999.1896.
5. Mao, J.D. and Schmidt-Rohr, K., *Separation of Aromatic-carbon 13C NMR Signals from Di-oxygenated Alkyl Bands by a Chemical-shift-anisotropy Filter.* Solid State Nucl. Magn. Reson. **2004**. 26(1) 36-45.DOI: 10.1016/j.ssnmr.2003.09.003.
6. Torchia, D.A. and Szabo, A., *Spin-lattice Relaxation in Solids.* J. Magn. Reson. **1982**. 49(1) 107-121.DOI: 10.1016/0022-2364(82)90301-8.
7. Schmidt-Rohr, K., *Simulation of Small-angle Scattering Curves by Numerical Fourier Transformation.* J. Appl. Crystallogr. **2007**. 40(1) 16-25.DOI: 10.1107/S002188980604550X.
8. deAzevedo, E.R., Hu, W.-G., Bonagamba, T.J., and Schmidt-Rohr, K., *Principles of Centerband-only Detection of Exchange in Solid-state Nuclear Magnetic Resonance, and Extension to Four-time Centerband-only Detection of Exchange.* J. Chem. Phys. **2000**. 112(20) 8988-9001.DOI: 10.1063/1.481511.
9. Reichert, D., Bonagamba, T.J., and Schmidt-Rohr, K., *Slow-Down of 13C Spin Diffusion in Organic Solids by Fast MAS: A CODEX NMR Study.* J. Magn. Reson. **2001**. 151(1) 129-135.DOI: 10.1006/jmre.2001.2337.
10. Bodenhausen, G., Freeman, R., and Turner, D.L., *Suppression of Artifacts in Two-dimensional J Spectroscopy.* J. Magn. Reson. **1977**. 27(3) 511-514.DOI: 10.1016/0022-2364(77)90016-6.

11. Jeener, J., Meier, B.H., Bachmann, P., and Ernst, R.R., *Investigation of Exchange Processes by Two-dimensional NMR Spectroscopy.* J. Chem. Phys. **1979**. 71(11) 4546-4553.DOI: 10.1063/1.438208.

12. Frisch, M.J., Trucks, G.W., Schlegel, H.B., Scuseria, G.E., Robb, M.A., Cheeseman, J.R., Scalmani, G., Barone, V., Petersson, G.A., Nakatsuji, H., et al., *Gaussian 16 Rev. B.02*. 2016: Wallingford, CT.

13. Lodewyk, M.W., Siebert, M.R., and Tantillo, D.J., *Computational Prediction of 1H and 13C Chemical Shifts: A Useful Tool for Natural Product, Mechanistic, and Synthetic Organic Chemistry http://cheshirenmr.info/.* Chem. Rev. **2012**. 112(3) 1839-1862.DOI: 10.1021/cr200106v.

14. Janani, H., Marxsen, S.F., Eck, M., Mecking, S., Tashiro, K., and Alamo, R.G., *Polymorphism and Stretch-Induced Transformations of Sustainable Polyethylene-Like Materials.* ACS Macro Lett. **2024**. 13(2) 201-206.DOI: 10.1021/acsmacrolett.3c00639.

15. Janani, H., Kramer, C.W., Boyd, N.R., Eck, M., Mecking, S., and Alamo, R.G., *Crystallization Rate Minima of Aliphatic Polyesters Type PE-X,Y in a Wide Range of Undercooling: Role of CH2 Sequence Length and Layered Crystallites.* Macromolecules **2025**. 58(11) 5688-5705.DOI: 10.1021/acs.macromol.5c00614.

16. Duan, P. and Schmidt-Rohr, K., *Composite-pulse and Partially Dipolar Dephased MultiCP for Improved Quantitative Solid-state 13C NMR.* J. Magn. Reson. **2017**. 285 68-78.DOI: 10.1016/j.jmr.2017.10.010.

17. Fritzsching, K.J., Mao, K., and Schmidt-Rohr, K., *Avoidance of Density Anomalies as a Structural Principle for Semicrystalline Polymers: The Importance of Chain Ends and Chain Tilt.* Macromolecules **2017**. 50(4) 1521-1540.DOI: 10.1021/acs.macromol.6b02000.
